# Identifying oral microbiome alterations in adult betel quid chewing population of Delhi, India

**DOI:** 10.1371/journal.pone.0278221

**Published:** 2023-01-04

**Authors:** Mayank Bahuguna, Sunila Hooda, Lalit Mohan, Rakesh Kumar Gupta, Prerna Diwan

**Affiliations:** 1 Department of Microbiology, Ram Lal Anand College, University of Delhi, South Campus, New Delhi, India; 2 Department of Biotechnology, Delhi Technological University, Rohini, Delhi, India; University of the Pacific, UNITED STATES

## Abstract

The study targets to establish a factorial association of oral microbiome alterations (oral dysbiosis) with betel quid chewing habits through a comparison of the oral microbiome of Betel quid chewers and non-chewing individuals. Oral microbiome analysis of 22 adult individuals in the Delhi region of India through the 16S sequencing approach was carried out to observe the differences in taxonomic abundance and diversity. A significant difference in diversity and richness among Betel Quid Chewers (BQC) and Betel Quid Non-Chewers (BQNC) groups was observed. There were significant differences in alpha diversity among the BQC in comparison to BQNC. However, in the age group of 21–30 years old young BQC and BQNC there was no significant difference in alpha diversity. Similar result was obtained while comparing BQC and Smoker-alcoholic BQC. BQ smoker-chewers expressed significant variance in comparison to BQC, based on cluster pattern analysis. The OTU-based Venn Diagram Analysis revealed an altered microbiota, for BQ chewing group with 0–10 years exposure in comparison to those with 10 years and above. The change in the microbial niche in early chewers may be due to abrupt chemical component exposure affecting the oral cavity, and thereafter establishing a unique microenvironment in the long-term BQC. Linear discriminant analysis revealed, 55 significant features among BQC and Alcoholic-Smoker BQC; and 20 significant features among BQC and Smoker BQC respectively. The study shows the abundance of novel bacterial genera in the BQC oral cavity in addition to the commonly found ones. Since the oral microbiome plays a significant role in maintaining local homeostasis, investigating the link between its imbalance in such conditions that are known to have an association with oral diseases including cancers may lead to the identification of specific microbiome-based signatures for its early diagnosis.

## 1. Introduction

The oral microbiome is a highly personalized and diverse microbiota in the human body playing a pivotal role in the health maintenance of the host [1,2]. Studies have highlighted microbial variation and its relation to various diseases, but there is a lack of knowledge on the onset of oral dysbiosis caused due to harmful masticatory products like Betel quid (BQ). The oral cavity is made up of diverse structures and tissues, including cheeks, gingiva, gingival sulcus, lips, teeth, tongue and palate, providing different habitats, nutrients, and growth conditions [3] for microbes. Changes in oral microbiota have been associated with different types of cancer [4], but whether those changes are the cause or the consequence of the pathological processes or are the external effects drawn from substance exposure such as BQ, leading to dysbiosis of primitive microbiome is still not clear [3].

Betel Quid (BQ), a preparation of betel leaf (*Piper betle*), areca nut (*Areca catechu*), and catechu (*Acacia catechu*) along with slaked lime, is consumed with or without tobacco [5]. The chemical components of these smokeless tobacco (SLT’s) forms mainly comprise polyphenols and alkaloids such as arecoline, arecaidine, guvacoline, guvacine, and tannins. These components have multifaceted effects on the oral cavity of a betel quid chewer (BQC) when compared to betel quid non-chewers (BQNC) including the changes in local microbiome.

Areca nut (AN) in BQ preparation is chewed by around 10–20% of the world’s population accounting for around 700 million individuals [6–8]. Areca nut is the most commonly used addictive substances in the world after tobacco, alcohol, and caffeine [9]. The prevalence of BQ chewing is high among the Indian population and this trend is also observed in other parts of South Asia and Melanesia [6]. BQ chewing has decreased in certain regions of Thailand but on the other hand, there has been a 72–80% rise in BQ usage in the west pacific region. In a previously reported Asian study, chewing rates among men were significantly higher in the neighbouring countries of India, such as Mainland China, Nepal, and Sri Lanka [9]. India being, one of the largest producers of BQ components worldwide is also one of its highest consumer [8]. Its use as a masticatory product is highly prevalent in the North East region of India as part of the cultural ethnicity, also accounting for a significant number of cancer cases associated with the use of SLTs as per recent data [10,11]. BQ is also popular as a home remedy across the Indian subcontinent due to its pain-relieving property, especially in toothaches, which has resulted in growing addiction among the lower class population in India [11]. It is also used in wide-ranging human diseases and disorders, including anemia, digestive disorders and infections, vitiligo or leucoderma, leprosy, dental infections, urinary infections as well as obesity. AN acts as a general stimulant [8]. Its component arecoline is a muscarinic acetylcholine receptor agonist, which initiates a cholinergic effect on the parasympathetic nervous system [12]. It also functions as a psychoactive agent inducing various responses, which acts as a stimulant for neurodegenerative responses, including an upsurge in sense of alertness, capacity to work for a longer time, diminished hunger, euphoria, palpitation, salivation, warmth, feeling of wellbeing, and easy digestion. Areca nut chewing, alcohol and smoking, together have been considered to be primary causes of oral cancer in the previously reported study by Zini et al. 2010 [13]. The alkaloid, arecoline is carcinogenic and reported to eventually transform the stem cells with carcinogenic properties [5,6,14,15]. It undergoes a nitrosation reaction giving rise to a diverse array of betel quid-specific nitrosamines (BSNAs) within the oral cavity, which could lead to a prevalent shift of acid-tolerant bacteria. Likewise, nitrosamine formation may also be facilitated by bacterial nitric oxide or other trace elements present in areca nut [16–18]. Other than nitrosation, arecoline also triggers other pro-carcinogenic alterations such as the production of reactive oxygen species, p53 inhibition, up-regulation of inflammatory cytokines, repression of DNA repair, and DNA damage [19,20]. Major polyphenols in areca nut including: arecoline, catechin, flavonoids, and leucoanthocyanidin (flavan 3:4-diols) have been reported to exhibit pathological alterations after epithelial inflammation [21]. Betel leaf, another component of BQ contains (hydroxychavicol), a phenolic component that could be detected in saliva at 4.6mM concentration after betel quid chewing [22]. Slaked lime in BQ, may alter intracellular calcium homeostasis, and excess calcium may bring some changes in the oral microbiota [23]. Various investigations have been done to comprehend and explain the relationship between BQ chewing habits with associated substances and patterns in various locales through study of histological changes displayed a relation to cancer progression known as precancerous lesions [8,24–26]. Some studies also have reported alcohol abuse along with BQ chewing, affects the microbiome balance leading to oral dysbiosis, and developing the risk of oral cancer in habitual chewers [27,28]. Alcohol may cause damage to epithelial cells, increasing the permeability of the epithelial cell membrane, which facilitates the penetration of carcinogens into the epithelium [27–29]. In previously done investigations, it is reported that alcohol and smoking habits with betel quid chewing might alter oral microbial compositions [30,31]. BQ along with smoking also modulates cigarette carcinogen benzo[a]pyrene-mediated toxic effects through dihydrodiol dehydrogenase (DDH) and hypoxanthine phosphoribosyltransferase (HPRT) induction [32].

BQ chewing potentially influences oral microbiota epidemiologically in the population [7,8,33,34]. Strong evidence is available for the role of *Porphyromonas gingivalis*, as a biomarker for oral cancer, and oral squamous cell carcinoma (OSCC) [35]. Oral bacteria can infect hard and soft tissues majorly observed in dental caries and periodontitis, additionally by the rupture of epithelial cells through BQ chewing. Its toxicity mainly depends on the chewing exposure and its duration [36,37]. Similar effects of BQ products leading to dysbiosis are also enhanced by various local cultural practices, lifestyle choices, and its abundant easy availability at a cheap cost. These initiations to such harmful oral diseases finally leading to oral cancer appear to be a major factor at the base level for the dysbiotic changes which in turn may slowly precede the change in the microbiota of an individual’s oral microbiome. In previous studies [38] the high prevalence of BQ chewing has shown to lead to Oral submucosal fibrosis (OSF), which later develops into OSCC [39].

Oral microbiota compositional changes have recently been found linked to oral cancer, where a colonization shift in tumor and non-tumorous site are distinguishable. The oral metagenomic data can be analyzed [40] and compared to databases such as the Human Oral Microbiome Database (HOMD) and NCBI which provides more than 700 phylotypes of bacteria, residing in the human oral cavity. The 12 major phyla reported in the oral sites include *Firmicutes*, *Fusobacteria*, *Proteobacteria*, *Actinobacteria*, *Bacteroidetes*, *Chlamydiae*, *Chloroflexi*, *Spirochaetes*, *SR1*, *Synergistetes*, *Saccharibacteria* (TM7), and *Gracilibacteria* (GN02) [41]. A study by (Pushalkar et al.) displayed bacterial species of *Streptococcus sp*. *oral taxon 058*, *Peptostreptococcus stomatis*, *Streptococcus salivarius*, *Streptococcus gordonii*, *Gemella haemolysans*, *Gemella morbillorum*, *Johnsonella ignava*, and *Streptococcus parasanguinis* reported as highly associated with tumor site in terms of prevalence [42]. In a stage-specific study of OSCC patients, a relative abundance profile for Fusobacteria was observed in oral cancer progression with comparison to healthy controls (2.98%) to OSCC stage 1 (4.35%) through stage 4 (7.92%). As a checkpoint, a lower relative abundance was reported for the genera of Streptococcus, Haemophilus, Porphyromonas, and Actinomyces. *Fusobacterium periodonticum*, *Parvimonas micra*, *Streptococcus constellatus*, *Haemophilus influenza*, and *Filifactor alocis* were certain bacteria that were detected also in the cases of cancer-associated diseases like periodontitis and dental caries [30].

Studies have shown an association between BQ and carcinogenesis and recent reports are linking oral microbiome alterations with cancer [31]. However, dysbiotic changes involving BQ-induced carcinogenesis largely remain unexplored. Therefore, the present study aims to identify the taxonomic compositional changes in the oral microbiome through 16S rRNA sequencing and community structure variance in BQ chewers and non-chewers. This will facilitate the understanding of whether the chewing and associated substances will lead to dysbiosis which may further be associated with progression of oral diseases including cancer [1,43,44]. This approach may also lead to identification of potential strategies for early detection of precancerous changes and apply this information in developing consortia of microbes, which can reverse the changes in the oral cavity of an individual through probiotic-based products.

## 2. Materials and methodology

### 2.1 Study population

The study was approved by Institutional Ethics Committee (IEC) and consent taken from participating sample population. The information was obtained through a questionnaire. A total of 32 individuals (28 males and 4 females) pursuing different occupations were identified. The study groups were divided into 3 categories: BQ chewers (BQC)—Habitual individuals for BQ chewing, non-chewers (BQNC)–Individuals with no history of BQ chewing, and Occasional/previous chewers (BQOC)–Having a frequency of BQ chewing either once in a couple of months or having a previous history of chewing as confirmed from the participant after confirmation of chewing habits.

### 2.2 Data and oral sample collection

A structured questionnaire was presented explaining the purpose of the study. The information was inquired for demography, BQ chewing with or without smoking and alcohol consumption, BQ chewing habit (duration and frequency of consumption), and antibiotic intake.

Samples from different sites of the oral cavity including tongue surface, hard and soft palate, subgingival region, cheeks, and buccal mucosa were collected under the supervision of a dental clinician and research staff and suspended in 1ml sterile saline solution (0.85% NaCl) provided in Himedia Transport Swab Kit (http://surl.li/aeapx). All samples were processed immediately. The cotton swab was swirled and cut down inside the tube followed by a short cycle of vortexing. The remaining material from the swab was procured by pressing against the walls of the microcentrifuge tube to extract the exfoliated cheek cells as well as other tissues into the saline solution. After getting the material of swabs dislodged into the saline, extracted volumes were pooled 6 times by collecting 6 different swabs from each individual. The pooled saline was centrifuged at 10,000g for 10 mins to pellet down the cells and other tissues. A final volume of 1ml was processed further, and the remaining supernatant was discarded. The DNA extraction was carried out following the processing protocol of QIAamp DNA Microbiome Kit (QIAGEN) www.qiagen.com/hb-1792 with minor modifications under a controlled condition. The modification included: Substitution of tissue lyser step with vortexing the reaction mix for 10 minutes at high speed. The samples were stored at -20°C until sent to the 16S rDNA sequencing facility to obtain the raw sequence data.

### 2.3 16S rDNA sequencing

The total DNA concentration of the samples was checked by NanoDrop 2000 spectrophotometer for QC criteria fulfillment, and extracted DNA was pooled for performing PCR and further, sequencing. The assay targeted the V3-V4 region of the bacterial 16S ribosomal RNA gene, by utilizing barcoded universal primers V13F and V13R with a sequence of (5’ AGAGTTTGATGMTGGCTCAG 3’ for forward and 5’ TTACCGCGGCMGCSGGCAC 3’ for reverse primer) [45]. The amplicons generated were purified with ampure beads (removal of unused primers) and 8 cycles of PCR were performed additionally using Illumina barcoded adapters for preparation of sequencing libraries. Quantification was done through Qubit dsDNA High Sensitivity assay kit (https://www.thermofisher.com/order/catalog/product/Q32851#/Q32851) followed by sequencing using Illumina Miseq with a 2x300PE v3 sequencing kit (http://surl.li/aeare) to generate 0.2 to 0.5 million paired-end reads (Biokart India).

### 2.4 Data processing and statistical analysis

The sequence data analysis was carried out against the NCBI and eHOMD databases. The bcl data was de-multiplexed into.fastq raw data format. The de-multiplexed data quality was checked using Fastqc (Version 0.11.9) and Multiqc (Version 1.10.1) tools followed by trimming of adapters and low-quality reads by TRIMGALORE (for workflow refer to, S17 Fig). The QC passed samples qualified for further analysis were processed by (Biokart Pipeline) for 16S metagenomics studies. After finishing the run, the raw OTU table was obtained followed by clustering utilizing reference based approach with 97% similarity threshold and further processed for visualization. The abundance feature tables and the top ten genera in each sample were constructed using Microsoft excel (2010). Further, Alpha diversity, Beta diversity, PCoA plot, LEfSe, etc were done using Microbiomeanalyst (online tool: https://www.microbiomeanalyst.ca/). The diversity measures were applied at the genus level to get the maximum out of the results obtained. The data was rarefied from the raw counts of the OTU table from the mentioned 16s metagenomic pipeline. In addition to other technical mentions, the community diversity profiling was primarily done by employing Vegan R packages in Microbiome Analyst [46]. Alpha diversity, the diversity present within a sample of a group was drawn based on the Shannon-diversity index (for the number of unique taxa and its richness) and the Shannon-evenness index (providing a relative abundance of unique taxa), which describes the actual diversity of a community. The outlier points from Shannon indices box plots were considered as samples presenting less diversity. The major concern of other statistical methods employed for the estimation of diversity depends on the analysis feasibility of our study. For ex- Chao1 statistical method, estimates the richness by including the number of rare organisms that may have been lost due to under-sampling. On the other hand, the observed genus explains the number of unique taxa present in a sample set.

Beta Diversity i.e., diversity differences (similarity or dissimilarity) between the two samples based on the dissimilarity matrix. This diversity analysis involved (a) How much similarity is present between samples using a non-phylogenetic Bray-Curtis index; (b) Dissimilarity matrix visualization in lower dimensions through Principal Coordinate Analysis (PCoA) in 2 D or 3-D plot format. Each point in the 3D plot represents the microbiome of a single sample within a microbial community. Each axis represents the variation percentage between samples, where the X-axis represents the highest dimension of variation and the Y-axis, the second-highest dimension of variation. Further, each point or sample displayed on PCoA plots is coloured based on the sample group of a specific feature. The statistical significance of the clustering pattern in ordination plots was evaluated using Permutational ANOVA (PERMANOVA). The criteria of PERMDISP explain a difference in dispersion (variance) between any of the groups, while ANOISM, explains the presence/absence or abundance of taxa present in a dataset, with a statistical significance.

Linear Discriminant Analysis (LDA) Effect Size (LEfSe) Plot which employs the Kruskal-Wallis rank-sum test was used for detection the features with significant differential abundance with regards to genus. The analysis evaluated the relevance or effect size of differential abundant features based on a p-value cut-off of 0.05 with the original filter and Log LDA score of 2.0. The test was used to analyse the abundant genera in a group compared to another group, for knowledge about the distribution of taxa/ organism among/ between the groups [47]. Unique OTU with differential comparison was done through an interactive online tool of Venn Diagram depiction (https://bioinfogp.cnb.csic.es/tools/venny/index.html) for comparing lists. It shows the percentage of unique organisms present in each sample and the percentage of organisms common to the pair and all the samples.

## 3. Results

### 3.1 Characteristic of the study population

32 oral samples were collected from people living in different parts of Delhi, India along with their information about demography, BQ chewing exposure, associated substances (smoking-alcohol) consumption, and gender. The final sample set analysed constituted 20 males and 2 females, where 9 were Current chewers (BQC), 9 Non-chewers (BQNC), and 4 with either former history of chewing or Occasional chewers (BQOC). Furthermore, 4 BQC individuals additionally had the habit of Smoking and 2 BQC individuals had an association with alcohol along with smoking (Table 1). The sequencing analysis filtered out 10 low-quality reads, removed chimeras, and normalized all samples for comparative analysis.

**Table 1:** Characteristics of study individuals (n = 22).


Categories		Number of Participants
**Category I**	**Chewers versus Non-chewers and Occasional (Overall Population)**	
	Total individuals	18
	Betel quid chewers	9
	Betel quid non-chewers	9
**Category II**	**Age range group [21–30 years] Chewers Vs. Non-chewers**	
	Total individuals	8
	Betel quid chewers (21–30 years Age)	4
	Betel quid non-chewers (21–30 years Age)	4
**Category III**	**Sex difference group [Males] Chewers Vs. Non-chewers**	
	Total individuals	12
	Betel quid chewers (Males)	6
	Betel quid non-chewers (Males)	6
**Category IV**	**Associated Substances grouping Chewers Vs. Alcoholic-smoker Chewers**	
	Total individuals	8
	Betel quid chewers	4
	Betel quid chewers (Alcoholics-Smokers)	4
**Category V**	**Smoking associated grouping Chewers Vs. Smoker Chewers**	
	Total individuals	8
	Betel quid chewers	4
	Betel quid chewers (Smokers)	4
**Category VI**	**Chewing history grouping [In association with betel quid]**	
	0–10 years	5
	10 years and above	5

### 3.2 Oral bacteriome taxonomic classification

The oral specimens of the 22 study subjects generated a total of 11,136,729 raw sequences and 11,058,370 quality-filtered sequences. Category-wise, BQC, BQNC, and BQOC yielded an average of 450538 (std. dev. 2,29,961), 549301 (std. dev. 1,99,667), and 514951 (std. dev. 1,30,212) quality-filtered sequences respectively, with a mean length of 528 bp. Three samples yielded ≤ 0.1 M sequences and were excluded for diversity and taxonomic comparisons. The complete sequencing dataset was submitted to the National Centre for Biotechnology Information (NCBI) [Bio sample Accession number- PRJNA853176]. A total of 3,129,170 reads with an average count of (1,42,235), were identified among 1233 OTUs in a sample set of 22 individuals after filtering with an approximate (0.03–0.5%) default threshold. Overall, 100% OTUs were classified at the phylum level, 77.21% at the genus level, and 58.10% were observed at the species level. With eHOMD database, for human Oral microbiome provided a total OTU count of 1286 post-filtration, with a default threshold of (0.75%). Taxonomic levels below phylum and above genus, e.g., class, order, and family, were not included in the criteria. Considering the whole sample population data based on NCBI; 14 bacterial phyla were identified among which, the most abundant ones, based on relative abundance with a threshold value of (1%<) were Firmicutes with the highest percentage (38.64%), followed by Proteobacteria (29.02%), Bacteroidetes (24.78%), Actinobacteria (4.16%), and Fusobacteria (2.58%). While, less abundant phyla included Acidobacteria (0.22%), Chloroflexi (0.17%), Planctomycetes (0.14%), Gemmatimonadetes (0.09%), Verrucomicrobia (0.04%), Ignavibacteriae (0.03%), Cyanobacteria, Candidatus Saccharibacteria, and Spirochaetes (0.02%) (S1 Fig). *Prevotella* (15%) was the most abundant among the 78 identified genera, followed by *Bacteroides* (12%), *Streptococcus* (11%), *Neisseria* (10%), *Haemophilus* (9%) and *Lactobacillus*, *Fusobacterium and Acetobacter* (3%) based on the threshold value of (1% <) for determining abundance factor. *Rothia*, *Bacillus* (2%), and *Geosporobacter* (1%) were low in abundance, among others (S2 Fig). An overall count of 75 species was recorded including *Prevotella denticola* (17%), *Streptococcus mutans* (11%), *Neisseria animalis* (10%), *Bacteroides dorei* (8%), *Haemophilus parainfluenzae* (6%) and *Fusobacterium nucleatum* (4%) (S3 Fig). 30 genera were identified with unclassified as well as classified species, among which *Bacteroides* (an average of 21%) was the most abundant, followed by *Streptococcus* (14%), *Acetobacter* (12%), *Prevotella* (10%), *Haemophilus* (8%), and *Neisseria* (6%). While the low abundant ones included *Rothia*, *Leuconostoc*, *Bacillus*, *Pediococcus*, *Arachidicoccus* (1%) respectively (S4 Fig). The two genera not classified at the species level were *Cycloclasticus* and *Lachnoclostridium* which could be a unique lead to unidentified bacteria up to species level in the oral samples collected.

With eHOMD 93% OTUs were identified at the phylum level, 78.79% at the genus level, and 39.82% observed at the species level. Considering the phylum level classification, 4 bacterial phyla were identified among which, Firmicutes was the most abundant, based on threshold value of (1%<) followed by Bacteroides (23%), Proteobacteria (21%), and Actinobacteria (6%). In case of BQ chewers, and BQNC a similar trend was observed. While BQOC, displayed Proteobacteria with (29%) as the 2nd relatively abundant phylum (S14 Fig).

#### Groupwise classifications based on NCBI database

**Fig 1:**
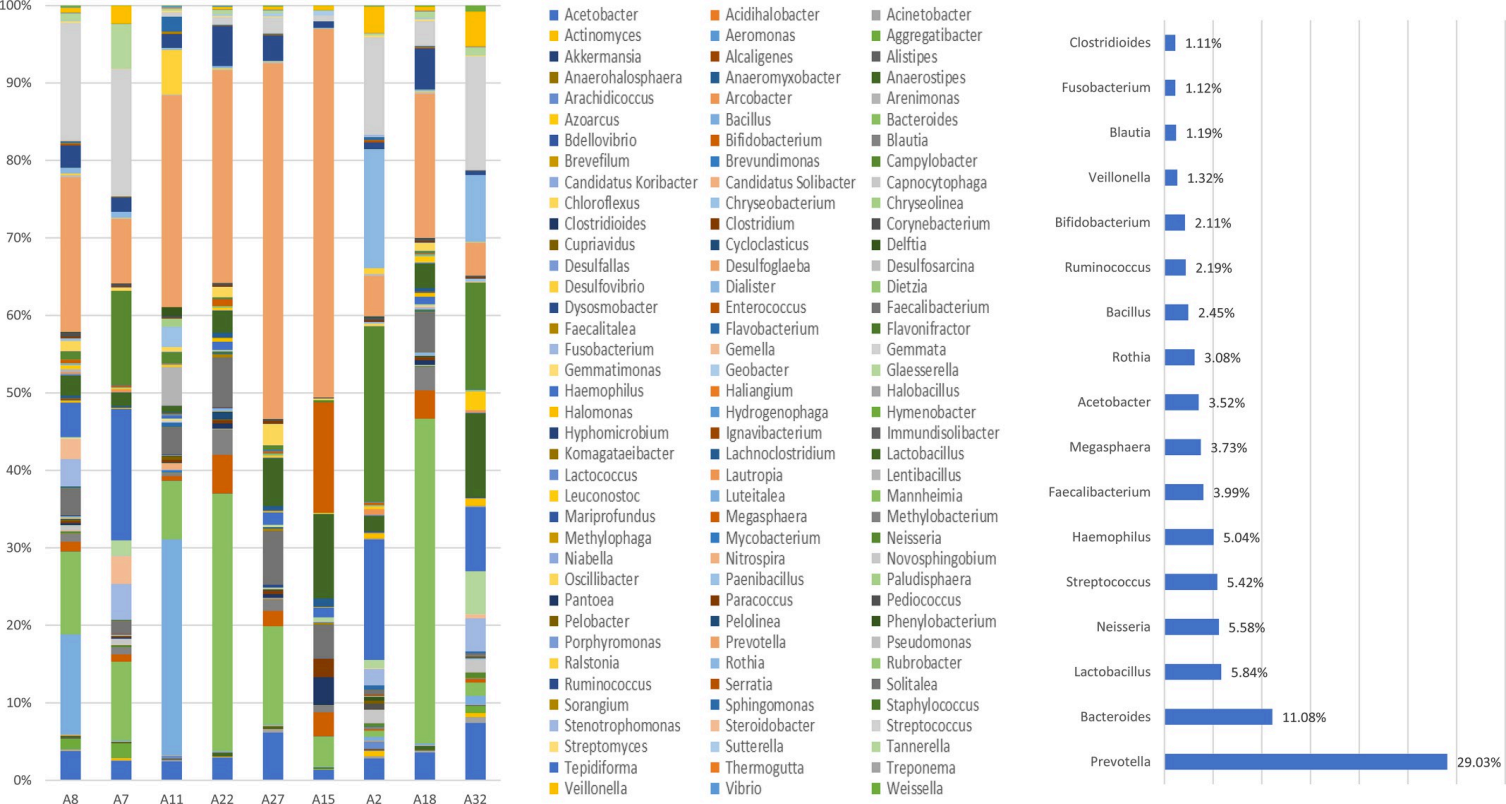
**a.** Genus-level oral microbiome composition of Betel quid Chewers: 114 genera including *Prevotella* (29%), *Bacteroides* (11%), *Lactobacillus*, *Neisseria*, *Streptococcus*, and *Haemophilus* (5%); **b.** Abundant genera detected in the Betel quid chewer individuals (excluding genera detected in lower than 1% of relative abundance among the samples).

A total of 16 phyla, 114 genera, and 108 species were identified in the BQC group. Out of the 16 phyla, Firmicutes was the predominant phylum comprising 41% OTU counts followed by Bacteroidetes (31%), Proteobacteria (20%), Actinobacteria (4%), and Fusobacteria (1%). *Prevotella* was the most abundant genus (based on percent relative abundance, among the OTU assigned) comprising 29% of taxa followed by *Bacteroides* (11%), *Lactobacillus*, *Neisseria*, *Streptococcus*, and *Haemophilus* (5%), and *Faecalibacterium* (4%), which were detected in all BQC (Fig 1A and 1B). At the species level, the most abundant ones included: *Prevotella denticola* (33%), *Bacteroides dorei* (7%), and *Fecalibacterium prausnitzii* (5%). Two *Prevotella* species were identified including *Prevotella denticola* and *Prevotella melaninogenica*. The abundance variation observed for *Prevotella* species ranged from 0.52% for *Prevotella melaninogenica* to 33% for *Prevotella denticola*.

**Fig 2:**
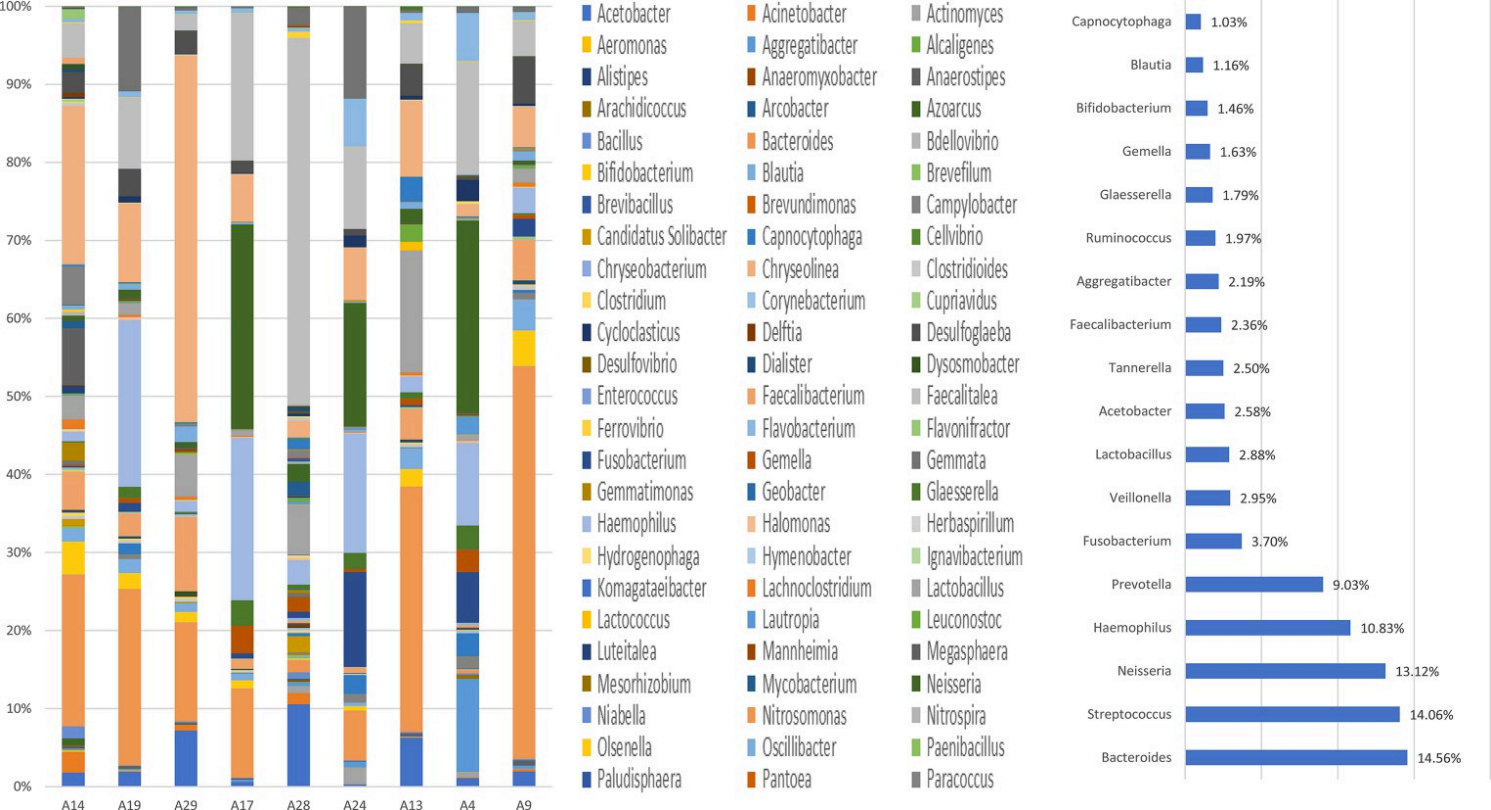
**a.** Genus-level oral microbiome composition of Betel quid Non-Chewers: 102 genera including *Bacteroides* (14.56%), *Streptococcus* (14%), *Neisseria* (13%), *Haemophilus* (10%), and *Prevotella* (9%); **b**. The abundant genera detected in the Betel quid non-chewer individuals (excluding genera with less than 1% of relative abundance).

Among BQNC, a total of 15 phyla, 102 genera, and 99 species were identified in the oral samples. Among the 15 phyla, Firmicutes predominantly comprised 38% of OTU counts followed by Proteobacteria (31%), Bacteroidetes (22%), and Nitrospirae, Actinobacteria, and Fusobacteria (3.3%). *Bacteroides* was the most abundant genera (based on percent relative abundance, among the OTU) comprising 14.56% of taxa followed by *Streptococcus* (14%), *Neisseria* (13%), *Haemophilus* (10%), *Prevotella* (9%), *Fusobacterium* (3%), and *Veillonella*, *Lactobacillus*, *Acetobacter*, and *Tannerella* (2.5%) which were detected in all the individuals of Betel quid non-chewers (Fig 2A and 2B). At the species level, the highest abundant taxon comprised *Streptococcus mutans* and *Neisseria animalis* (13%), *Bacteroides dorei* (10%) followed by *Prevotella denticola* (9%). Six *Bacteroides* species were identified including *Bacteroides dorei*, *Bacteroides uniformis*, *Bacteroides fragilis*, *Bacteroides caccae*, *Bacteroides vulgatus*, and *Bacteroides salanitronis*. The abundance variation observed for *Bacteroides* species ranged from 0.0066% for *Bacteroides salanitronis* to 10% for *Bacteroides dorei*.

A comparative analysis was also done to compare the top 3 abundant phylum based on NCBI and eHOMD database among the BQC and BQNC. The groups displayed the differences in the Log transformed count graph, on the basis of median shift observation, for Proteobacteria and Firmicutes, but the p-value reporting for the intergroup comparison was non-significant (0.05<p-value) (S15 and S16 Figs).

**Fig 3:**
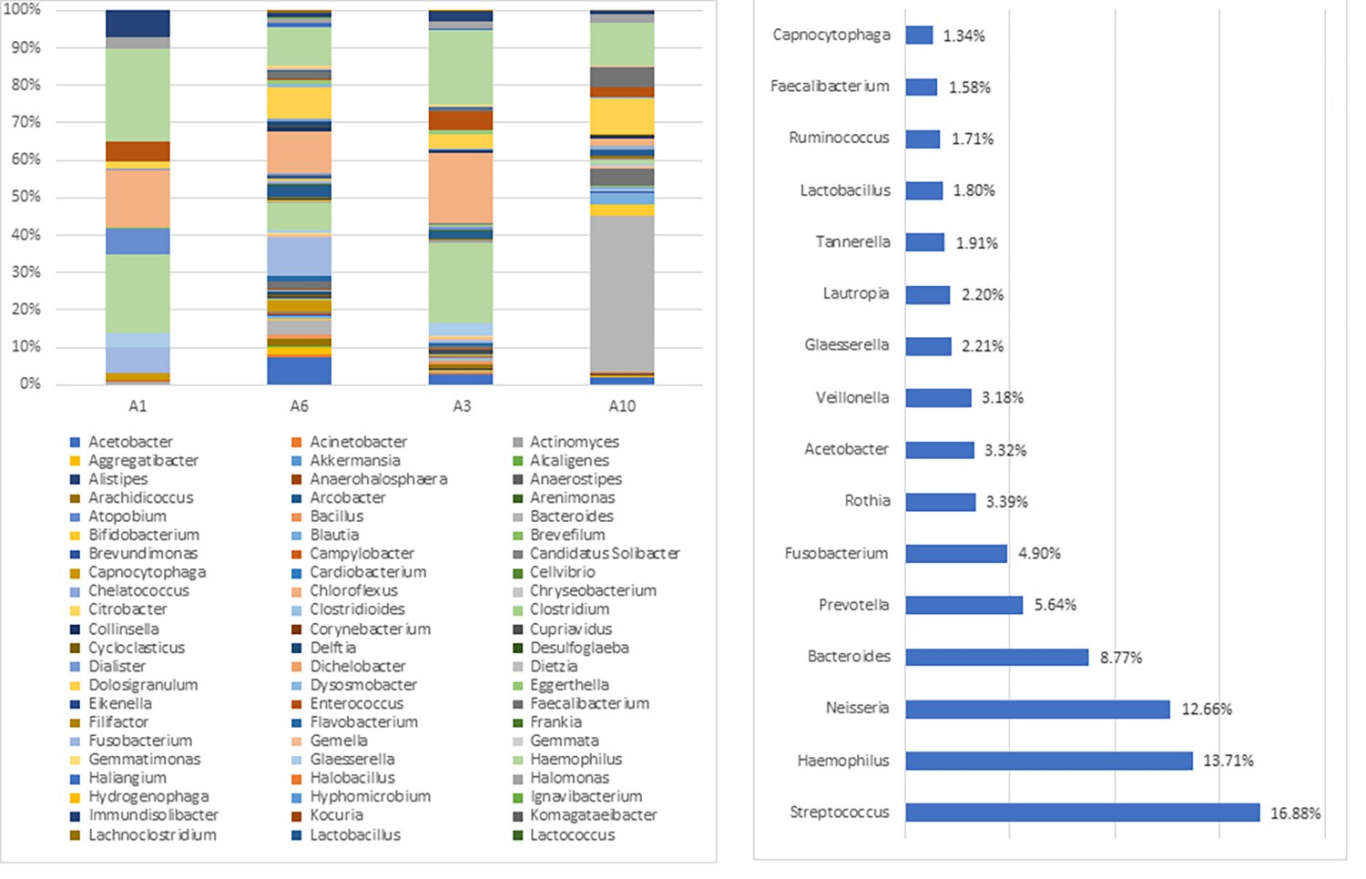
**a.** Genus-level oral microbiome composition of Occasional: 119 genera including *Streptococcus* (16%), *Haemophilus* (13%), *Neisseria* (12%), *Bacteroides* (8%), and *Prevotella* (5%); **b.** Abundant genera detected in the Betel quid Occasional chewer individuals (excluding genera with less than 1% of relative abundance).

A total of 16 phyla, 119 genera, and 128 species were reported in the BQOC group oral samples. In a total of 16 phyla, Proteobacteria was predominant comprising 38% of OTU counts followed by Firmicutes (34%), Bacteroidetes (16%), and Actinobacteria (4%). *Streptococcus* was the most abundant genus (based on percent relative abundance and OTU assigned) comprising 16% of taxa followed by *Haemophilus* (13%), *Neisseria* (12%), *Bacteroides* (8%), *Prevotella* (5%), and *Fusobacterium* (4%), *Rothia*, *Acetobacter*, and *Veillonella* (2%) and *Lactobacillus* (1%) all of which were detected in all the individuals of BQOC group (Fig 3A and 3B). At the species level, the highest abundant species observed were *Streptococcus mutans* (16%), *Neisseria animalis* (13%), *Haemophilus parainfluenzae* (11%), *Fusobacterium nucleatum*, and *Bacteroides dorei* (6%). Three *Streptococcus* species were identified including *Streptococcus mutans*, *Streptococcus pneumoniae*, and *Streptococcus salivarius*. The abundance variance pattern was observed for *Streptococcus* species ranging from 0.004% for *Streptococcus salivarius* to 16% for *Streptococcus mutans*.

### 3.3 Alpha and beta diversity comparisons in betel quid chewers and non-chewers

Based on the total number of bacterial sequences, **a significant difference** was observed between BQC (mean 4,50,538; std. dev. 2,29,961); BQNC (mean 5,49,301; std. dev. 1,99,667) (p = 0.041108), displaying more diversity and richness.

#### Alpha diversity

Alpha diversity was estimated through the Shannon diversity index. The results showed a positive difference for genera among BQC and BQNC based on the T-test statistic that checks whether the two means (averages) are reliably different from each other/not, thus explaining variance within the group. Therefore, a higher t-value signifies the difference in the genera inside the group and vice-versa. The T-test stat (0.364679) and p-value (0.726118) depicted the presence of a positive difference in genera in the analysis of the group comparison of BQC and BQNC (Table 2).

**Table 2:** Alpha diversity (comparison chart).

Grouping	T-test stat	p-value
**Betel quid Chewers/Non-chewers**	0.36	0.72
**Age range group [21–30 years] Chewers/Non-chewers**	- 0.91	0.45
**Sex difference group [Males] Chewers/Non chewers**	0.004	0.99
**Associated products grouping Chewers/Chewers** **[Alcohol and Smoking association]**	-5.05	0.03
**Smoking associated grouping Chewers/Chewers** **[Smoking association]**	2.37	0.14
**Chewing Time exposure group** **[Chewers in association to chewing substances]**	1.73	0.18

Based on age group, young individuals 21–30 years displayed less difference among the genera in BQC and BQNC as signified with a negative T-test stat value of (-0.919483) and confirmed with a p-value (0.4549096), that there is more than 45% chance for this pattern occurrence in further analysis. However, a slight positive difference in diversity was found among Male BQC Vs. Male BQNC, with a T-test stat of (0.004298), and p-value (0.996776). In subjects consuming associated substances besides BQ, a T-test stat (-5.05042) and p-value of (0.037041) was observed for BQC and BQC [Smoking-Alcohol association], displaying a negative difference but with a pattern reoccurrence of 3%; whereas a t-test value of (2.374983) with p-value (0.140792) for BQC and Smoker BQC represent a high level of difference between these categories at the genus level.

In samples tested with respect to lengths of chewing time of BQ, a positive difference at the genus level with a p-value (0.181089) and a T-test stat (1.735327). Therefore, an overall significant difference was observed among the majority of the groups; except 21–30 years old young BQC and BQNC, and BQC and BQC (Alcohol-Smoking Associated) at the genus level with a less difference of genera based on the Shannon-index diversity measure.

#### Alpha diversity- differential diversity measures basis

Alpha diversity measured through the Shannon-evenness index indicated a higher relative abundance for unique genera in the entire study population, with a 95% filtered diversity value threshold. Among BQC in comparison to the BQNC group, a significantly higher presence of unique taxa was seen based on Observed Genus (p = 0.05). However, a lower or nearly no unique taxa presence was observed in BQC as compared to BQNC in the age group of 21–30 years old (young individuals), based on Shannon (p = 0.45), Chao1 (p = 0.46), Observed genus (p = 0.49) (Table 3).

**Table 3:** Comparison of within-sample (alpha) diversity to bacterial microbiome based on the study population.

	Diversity Measures											
	Shannon				Chao1				Observed Genus			
Grouping	Mean	Standard Deviation	t-stat	p-value	Mean	Standard Deviation	t-stat	p-value	Mean	Standard Deviation	t-stat	p-value
**Betel quid Chewers Vs. Non-chewers**												
Chewers	2.47	0.33	0.36	0.72	145.95	8.69	0.50	0.62	129.77	16.55	2.30	0.05
Non-Chewers	2.48	0.20			140.27	10.04			122	22.05		
**Age range group [21–30 years] Chewers Vs. Non-chewers**												
Betel quid Chewers	2.48	0.21	-0.91	0.45	157.10	7.35	0.89	0.46	145	6.96	0.82	0.49
Betel quid Non-chewers	2.51	0.21			131.45	5.14			123.75	15.89		
**Sex difference group [Males] Chewers Vs. Non-chewers**												
Betel quid Chewers (Males)	2.47	0.33	0.004	0.99	138.14	8.60	-1.08	0.33	122.83	16.05	-0.07	0.94
Betel quid Non-chewers (Males)	2.48	0.20			136.61	7.93			122.5	20.14		
**Associated products grouping: Chewers Vs. Chewers with alcohol and smoking association**												
Betel quid Chewers	2.77	0.01	-5.05	0.03	165.56	6.18	-1.70	0.23	155.25	17.35	-0.12	0.91
Betel quid chewers with alcohol + smoking habit	2.30	0.009			154.78	5.60			145.75	1.29		
**Smoking associated grouping: Chewers Vs. Chewers [Smoking association]**												
Betel quid Chewers	2.76	0.02	2.37	0.14	161.69	10.57	-0.27	0.80	141.5	14.51	-0.93	0.44
Betel quid Chewers (Smokers)	2.44	0.37			141.80	9.68			124.75	20.75		
**Chewing Time exposure group [Chewers in association to chewing substances]**												
0–10 years	2.72	0.45	1.73	0.18	189.84	10.42	-2.65	0.07	167.40	24.33	3.14	0.05
10 years and above	2.61	0.23			184.77	13.41			154.60	12.40		

When comparing male BQC and BQNC, alpha diversity was found to be significantly lower in BQC based on Shannon (p = 0.99), explaining a low or no unique taxa presence at the genus level. BQC group displayed a higher alpha-diversity in terms of unique taxa at the genus level, described by Shannon (p = 0.03) in comparison to BQC with smoking and alcohol association. A trend of lower unique taxa was explained based on Shannon (p = 0.14) concerning the alpha diversity between the BQC and smoker BQC. Individuals with a chewing exposure history of 0–10 years displayed a higher alpha diversity as compared to individuals with more than 10 years of chewing history, based on the Observed genus (p = 0.05).

#### Beta diversity

Beta diversity i.e. in between-group/samples diversity observed in the ordination plots was evaluated through Permutational ANOVA (PERMANOVA). The f-value is used to determine the variance between the samples. The null hypothesis is considered to be equal to diversity and the alternate hypothesis to be the difference in PCoA plot patterns. To confirm this hypothesis, the p-value helps to test the significance, whereas the R-squared value calculates the amount of variation.

Among BQC and BQNC, PCoA plots demonstrated a strong clustering pattern. The plot was constructed as per the Bray-Curtis index, inferring a non-significant 5% variance with a (p < 0.41) PERMANOVA, which differs from that of ANOISM (p < 0.50) displaying a 0.5% of diversity. A similar level of low variance was also observed in the case of Jaccard index, with only a 4% diversity (p < 0.55) for PERMANOVA (S5A Fig) (Table 4A). Young individuals of 21–30 years displayed a clustering pattern within the groups of BQC and BQNC. The values based on the Bray Curtis index showed more than 17% variance in the samples(p < 0.23), while a difference of 4% variance is observed in ANOISM (p < 0.43). A differential variance was observed in the case of BQC evident through a single outlier point, while BQNC displayed none. With regards to the Jaccard index, a slight variation of 13% (p < 0.37) was observed in the case of PERMANOVA, while ANOISM expressed a similar percentage of 4% (p < 0.43) (S5B Fig).

**Table 4:** (a) Comparison of in between groups (beta) bacterial diversity based on the study population. (b) Comparison of in between groups (beta) bacterial diversity for the study population.

Grouping				Distance Methods		Statistical Methods							
						PERMANOVA		ANOISM				PERMDISP	
						F	p-value	R^c^		p-value		F	p-value
**Betel quid Chewers Vs. Non-chewers**													
Chewers			Non-Chewers	Bray-Curtis Index		0.91	< 0.41	-0.024		<0.502		0.10	0.74
				Jaccard Index		0.80	< 0.55	-0.024		<0.499		0.16	0.68
**Age range group [21–30 years] Chewers Vs. Non-chewers**													
Betel quid Chewers	Betel quid Non-Chewers			Bray-Curtis Index		1.30	<0.23	0.02		<0.433		0.05	0.82
				Jaccard Index		0.97	< 0.37	0.02		<0.433		0.03	0.85
**Sex difference group [Males] Chewers Vs. Non-chewers**													
Betel quid Chewers (Males)			Betel quid Non-Chewers (Males)	Bray-Curtis Index		1.80	< 0.13	0.11		<0.153		1.64	0.22
				Jaccard Index		1.32	< 0.18	0.11		<0.153		1.54	0.24
Grouping				**Distance Methods**	PERMANOVA			ANOISM			PERMDISP		
					F		p-value	R^c^	p-value		F		p-value
**Associated products grouping Chewers Vs. Chewers with alcohol and smoking association**													
Betel quid Chewers		Betel quid Chewers (Alcoholics)		Bray-Curtis Index	35.06		<0.041	1	<0.041		59		0.003
				Jaccard Index	19.63		<0.041	1	<0.042		77		0.001
**Smoking associated grouping Chewers Vs. Chewers [Smoking association]**													
Betel quid Chewers		Betel quid Chewers (Smokers)		Bray-Curtis Index	8.50		<0.034	0.77	<0.034		4.09		0.089
				Jaccard Index	5.41		<0.034	0.77	<0.034		6.37		0.044
**Chewing Time exposure group[Chewers in association to chewing substances]**													
0–10 years		10 years and above		Bray-Curtis Index	0.55		< 0.64	0.08	<0.594		0.0006		0.98
				Jaccard Index	0.60		< 0.75	0.08	<0.595		0.002		0.95

The male BQC group showed a variance of more than 15% in diversity (p < 0.13) (S6A Fig) and 1% of the variance in ANOISM (p < 0.15). A similarity matrix was evident with no outlier points observed; therefore, a strong correlation was inferred between the groups. While in the case of the Jaccard index, a slight variation of 11% (p < 0.18), was observed in the case of PERMANOVA, similar to ANOISM expressing 1% (p < 0.15). Bray Curtis Index displayed a variance of more than 85% diversity (p-value < 0.041) for BQ chewing associated with other habits, while ANOISM expressed a huge difference in a variance of 1% only with a (p < 0.041) (Table 4B) among BQC and Alcoholic-smoker BQC when considered. The PCoA plot displayed no correlation between the groups as indicated by a clearly separate zone verifying the variance value of 85% (S6B Fig). Individuals with smoking-associated habits, displayed a clustering pattern within the groups and expressed a variance of 58% diversity with (p-value < 0.03) as per the Bray-Curtis index, while ANOISM expressed a 59% of variance. With Jaccard Index, a 47% diversity difference was noted with a (p-value < 0.034). A higher variance was observed in the case of BQC with 2 outlier points, contributing to the group difference (Table 4B) (S7A Fig).

The BQ chewing history data provided an overall clustering pattern, and variation of only 6% in diversity for different chewing exposure, (p-value < 0.648) as per the Bray-Curtis index, while ANOISM drew only a 0.07% of the variation, (p < 0.594) (Table 4B). An average variance was observed in the case of 10 years and above individuals with 1 outlier point, displaying a lower diversity and a good clustering pattern expressed among the groups (S7B Fig).

### 3.4 Unique operational taxonomic elements

**Fig 4:**
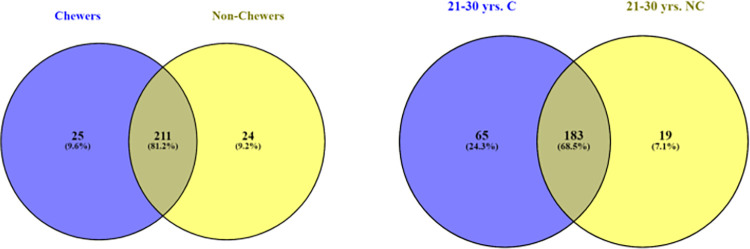
**a.** Genus-level oral microbiome Unique OTU elements composition of Betel quid chewers Vs. Non-Chewers; **b.** (Age Grouping) 21–30 years young individuals Betel quid Chewers Vs. Non-Chewers.

**Fig 5:**
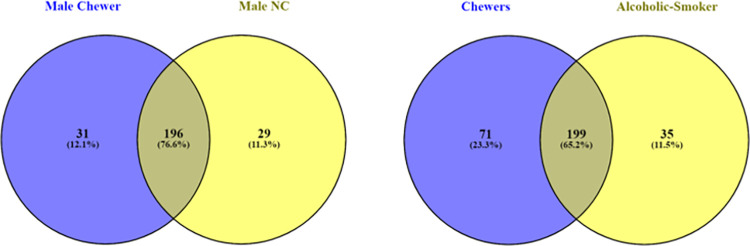
**a.** Genus-level oral microbiome Unique OTU elements composition of Betel quid Male chewers Vs. Non-Chewers; (Intra-Grouping); **b.** Betel quid Chewers Vs. Alcoholic-Smoker Chewer individuals.

**Fig 6:**
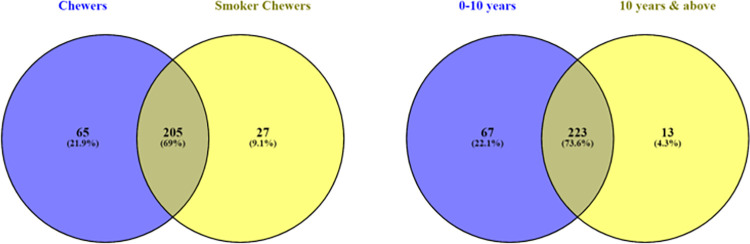
**a.** Genus-level oral microbiome Unique OTU elements composition of (Associated-Substance Grouping): Betel quid Chewers Vs. Smoker Chewers; **b.** Chewing History interval: 0–10 years, and 10 years-above chewing exposure time in years.

Unique OTU refers to the sequence count identifying the classified taxa subjected to clustering and correlation algorithm through Marker data profiling. Venn’s diagram was used to compare the list of genera for observing the unique taxon variance between BQC and BQNC. The data depicts (25) unique elements in BQC and (24) in BQNC (Fig 4A). Young individuals displayed a higher (65) unique count in BQC in comparison to BQNC (19) (Fig 4B). Among males, BQC displayed a slightly higher count of unique OTU (31) than BQNC (29) (Fig 5A). BQC showed a much higher unique OTU (71), as compared to Alcoholic-smoker chewers (35) (Fig 5B). BQ chewers displayed a higher count of unique OTUs (65) as compared to Smoker chewers (27) (Fig 6A). Chewing exposure history, also showed an altered microbiota, with a unique OTU count of (67) for a chewing exposure of 0–10 years and only (13) for 10 years and above chewing exposure (Fig 6B). This concludes a prominent change in microbiome of early chewers possibly due to the first exposure of components coming from BQ in the oral cavity, which stabilises with prolonged exposure, establishing a unique microenvironment build-up in long-term BQC.

### 3.5 Distinct taxon between the groups

**Fig 7:**
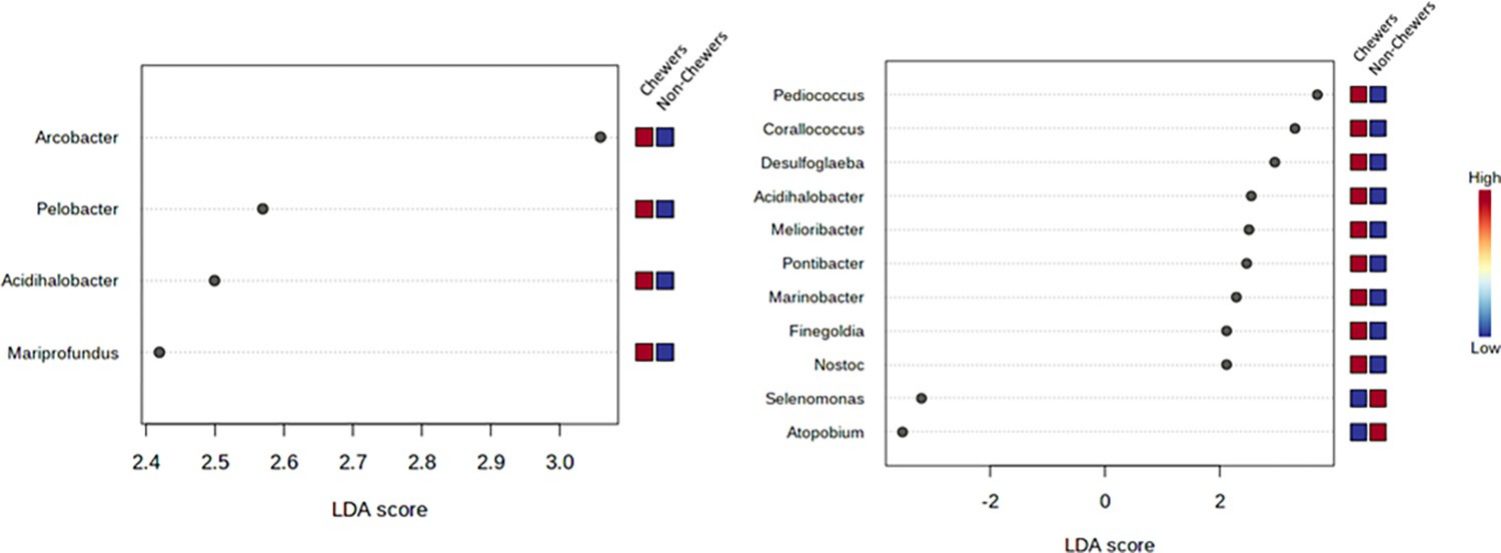
**a.** Lefse Plots: Genus-level oral microbiome significant features composition of Betel quid chewers Vs. Non-Chewers; **b.** (21–30 years old young individuals) Betel quid chewers Vs. Non-Chewers.

The bacterial communities in different groups were analyzed at the genus level by lefse plots. The abundance of a specific bacterial genus was compared to BQ chewing status. Significant different features were determined with a threshold value of (Original p*<*0.05) and a log LDA score of 2.0, for comparing the abundance of prevalent genera. The x-axis of the plot represents the effect size (LDA score) in the study population and colors represent in which group the genus was found to be more abundant compared to the other groups. The LDA score may be obtained as negative due to the order of numerator and denominator while calculating the effect size. The absolute values of the effect size interpret the scale difference between 2 groups for a certain taxon. In BQC Vs. BQNC, a total of (4) significant features were reported. The abundance was observed for *Arcobacter* genus detected in the oral cavity of BQC compared to that of BQNC, (p-value = 0.012) and LDA score of 3.06, expressing it to be significantly abundant (Figs 7A and S8A). In the case of 21–30 years age group, BQ chewing young individuals, an overall count of (11) significant features were observed among BQC and BQNC. A higher level of abundance was reported for *Pediococcus* detected in BQC, supported with (p-value = 0.043), LDA score of 3.69, in comparison to BQNC (Figs 7B and S8B).

**Fig 8:**
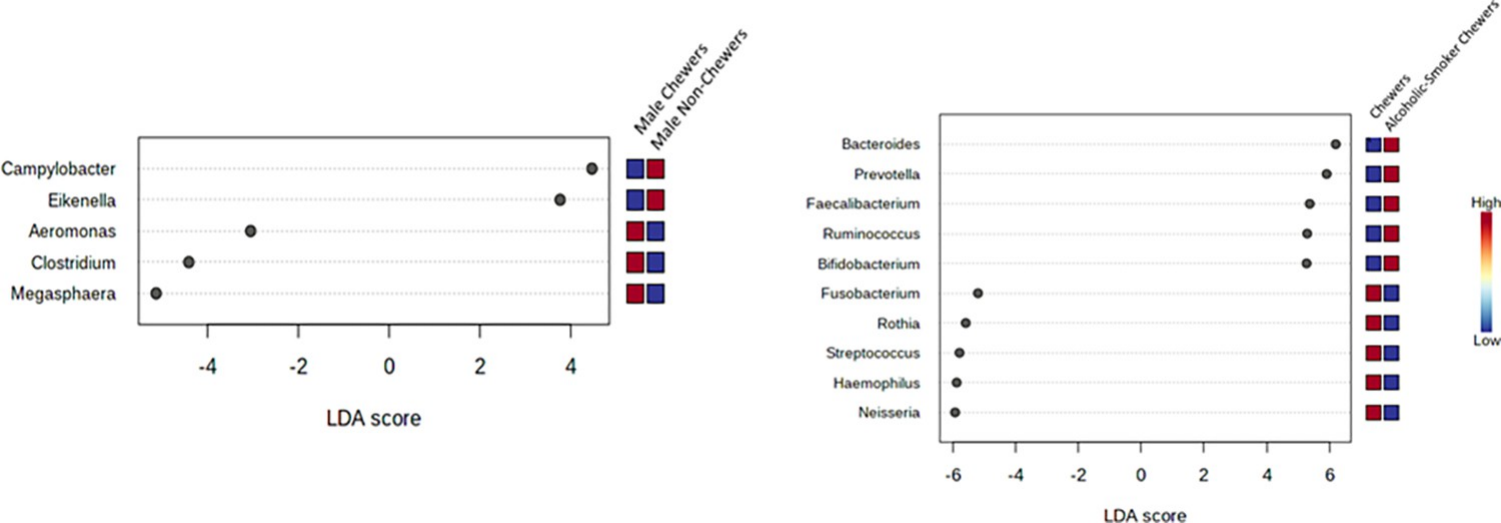
**a.** Lefse Plots: Genus-level oral microbiome significant features composition of Betel quid (Males) Chewers Vs. Non-Chewers; **b**. Betel quid Chewers Vs. Alcoholic-Smoker Chewers.

**Fig 9:**
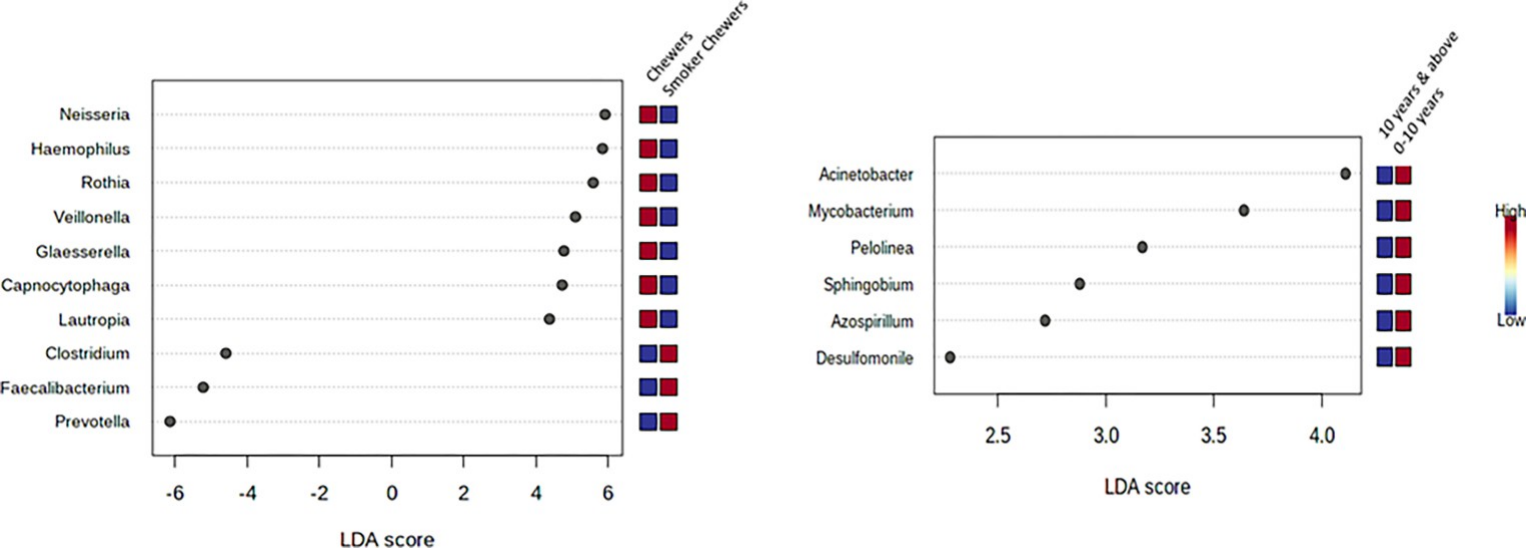
**a.** Lefse Plots: Genus-level oral microbiome significant features composition of Betel Quid chewer Vs. Smoker Chewers; **b**. Betel Quid Chewer (Chewing History).

When comparing male BQC with male BQNC 5 significant features were seen. The abundance was observed for *Campylobacter*, detected in BQNC with a p-value = 0.037 and LDA score of 4.47, verifying significant abundance (Figs 8A and S9A). While studying samples of associated substances with BQ chewing habit, 55 significant features among BQC and Alcoholic-Smoker BQC were recorded. The abundance was observed for *Bacteroides* detected in BQ alcoholic-smoker chewers (p-value = 0.020) and LDA score of 6.2, verifying significance with concern to BQC (Figs 8B and S9B). 20 significant features were observed among BQC and Smoker BQC with the highest abundance for *Neisseria* detected in BQC (p-value = 0.020 and LDA score of 5.92), verifying significant abundance (Figs 9A and S10A). With regards to BQ chewing history, an overall count of 6 significant features was observed with the abundance of *Acinetobacter* in the oral cavity of BQC with a chewing history of 0–10 years, in comparison to 10 years and above, (p-value = 0.036 and LDA score of 4.11) (Figs 9B and S10B).

The prevalence of organisms at the genus level is provided through core microbiome plots, to detect the threshold, based on abundance. LDA plots are thus, self-explanatory to the extent that they give us a clear idea about taxon which is highly present in one group, while observed vice-versa in the other group. Thus, BQC Vs. Alcoholic-smoker chewers displayed the highest significant feature count.

## Discussion

The present study was aimed at characterizing the diversity of oral microbiome in BQC and its comparison to BQNC and BQOC to identify specific microbial signatures. We found 14 bacterial phyla, 78 genera, and 75 species, of which 30 genera with unclassified species. Bacteroidetes, comprising *Prevotella* and *Bacteroides* genera make up a substantial part of the human oral microbiome. These exist in a complex mutualistic relationship degrading biopolymers (proteins and long-chain carbohydrates), facilitating the growth of other commensal and pathogenic microbial flora [48]. *Prevotella*, *Bacteroides*, and *Streptococcus* were the most abundant in all groups including chewers, non-chewers, and occasional chewers respectively (S11 Fig). The nutrient sources shapes the oral microbiome, exemplified by the rise in Betaproteobacteria and Fusobacteria with saturated fatty acid levels; sugars, and water-soluble vitamins with an abundance of Lactobacillaceae and Fusobacteria respectively [49,50]. Enterobacteriaceae, Proteobacteria, and Streptococcus have been found to be abundant in the oral cavity of the Indian population [51]. Similar results were obtained in the present study with Firmicutes- *Streptococcus*, *Lactobacillus*; Proteobacteria- *Neisseria*, *Haemophilus*, *Acetobacter*; Bacteroidetes- *Bacteroides*, *Prevotella*, and Fusobacteria- *Fusobacterium*; constituting a majority of the oral microbiome.

Several saccharolytic and aciduric species hasten the progress of OSCC and oral cancer, due to endotoxin release in the oral cavity including *Fusobacterium*, *Streptococcus*, and *Prevotella* species: *P*. *melaninogenica*, *P*. *intermedia*, *P*. *nigrescens*, and *P*. *veroralis* [51–55]. *Streptococcus*, *Prevotella*, and *Tannerella* are also notable oral pathogens, leading to various dental diseases such as periodontitis and dental caries [52,56,57]. *Bacteroides* (*B*. *gingivalis*, *B*. *intermedius*, *B*. *endodontalis*) are associated with destructive periodontitis, gingivitis, endodontal infections, and abscesses and are reported in the oral cavity of periodontal patients contrary in healthy subjects [58–60]. BQC having a long chewing history may form an oral abscess in the cavity, also hosting periodontal infectious *Bacteroides* leading to pockets formation. S. *pneumoniae*, *S*. *salivarius*, *and S*. *mutans* were identified in the oral cavity of BQOC. *However*, a decrease in *Streptococcus* genus was observed among BQC, contrary to the earlier observation by Hernandez et. al, 2017 [31] showing a 4-fold increase. *S*. *infantis*, a commensal oral microorganism [61] was found to increase considerably in BQC. *Streptococcus salivarius* has been shown to be significant in the cancer samples as compared to control samples [42,62] which can serve as a diagnostic marker along with *Prevotella* species for BQ association with OSCC [31,44,52,56].

Tobacco-specific nitrosamines (TSNA) and alcohol, has been reported to cause oral dysbiosis over prolonged usage contributing to the progress of oral cancer [26,63] changing profile from Gram-negative to Gram-positive bacteria resulting in high salivary acetaldehyde concentration [64,65] that gets readily absorbed. Oral bacteria such as *Streptococcus gordonii*, *S*. *oralis*, *S*. *mitis*, *S*. *salivarius*, and *S*. *sanguinis*, possessing the enzyme alcohol dehydrogenase (ADH), convert alcohol to acetaldehyde, hence playing an essential role in alcohol-related carcinogenesis in humans [56].

The commensal oral microbiome composition responsible for maintaining a healthy state may get affected by the incessant habit of BQ chewing. In BQC with chewing history from 0–10 years, the relative abundance of *Prevotella* increased; whereas it considerably decreased for *Haemophilus*, *Streptococcus*, and *Rothia*, while *Lactobacillus* was observed to be relatively stable. *Prevotella* followed by *Bacteroides* were the most abundant genera in the BQC group. *Bacteroides*, *Streptococcus*, *Neisseria*, *Haemophilus*, *Prevotella*, *Fusobacterium*, *Veillonella*, and *Lactobacillus* were found to be a major part of the commensal oral microbiota of BQNC, consistent with studies reporting healthy microbiota [61,66–70]. An increased abundance for *Prevotella*, *Lactobacillus*, *Faecalibacterium*, *Ruminicoccus*, and *Bifidobacterium* genera observed among the microbial community of BQC was in sync with the recent study by Uehara et. al, 2021 [44]. While, a decreasing pattern for *Bacteroides*, *Streptococcus*, *Neisseria*, *Haemophilus*, *Fusobacterium*, and *Veillonella* in BQC was observed (S12 Fig). 4 unique elements seen in BQC were *Megasphaera*, *Bacillus*, *Rothia*, *Clostridioides*, and *Tannerella*, *Aggregatibacter*, *Glaesserella*, *Gemella*, *Capnocytophaga* in BQNC.

With regard to alpha diversity, more unique taxa and a higher level of relative abundance was found in BQC as compared to BQNC at the genus level, and high evenness in BQC as compared to BQNC based on Observed genus indices (S13 Fig). Also, a higher level of diversity significance was observed for the groups of BQC and Smoker-Alcoholic BQC and differential chewing exposures, while the other 3 groups of Male BQC and BQNC, 21–30 yrs. BQC and BQNC, and Smoker BQC and BQC, displayed the least evenness.

Dealing with beta diversity, we observed less differences between BQC and BQNC with a strong clustering pattern (S5A Fig) as in case of male BQC and BQNC (S6A Fig). A slight difference in diversity is observed with outliers in the common zone of similarity, in 21–30 years young individuals and differential chewing exposure of BQ group. BQC vs. Smoker-alcoholic BQC and BQC with the smoking association in comparison to non-smoker BQC was observed with a noticeable differential matrix with a differential zone among the grouping samples (S6B and S7A Figs), validated with 85% and 58%) diversity respectively. Maximum unique OTUs were observed in the microbiome of 21–30 years BQC and BQNC, BQC Vs. smoker-alcoholic BQC, and among BQC Vs. smoker BQC individuals (Figs 4B, 5B and 6A). A nearly identical microbial community was observed in the oral cavity of BQC and BQNC in the overall population, male BQC and BQNC (Figs 4 and 5A). There was an abrupt rise in the unique oral community in early chewers, probably due to variations in pH and various other molecular and chemical changes which ensue, affecting the oral cavity (Fig 6b).

Lefse Plot encompasses distinction between the groups in terms of abundantly significant taxon [71]. Based on the LDA score and p-value, for the category BQC Vs. BQNC: significance was displayed by *Arcobacter* in BQC; for the category 21–30 years old BQC vs BQNC: 9 significant features out of 11 for BQC were observed with a trend above 2.0 LDA score. Some major phyla included *Pediococcus*, *Corallococcus*, *Desulfoglaeba*, etc. In case of Male BQC vs. BQNC, 5 significant features with (< -2.0) LDA score was observed for BQC: *Aeromonas*, *Clostridium*, *Megasphaera*. BQC Vs. Alcoholic-smoker BQC showed, 55 significant features with the presence of *Bacteroides* at a significant level in Alcoholic-smoker BQC. In relation to associated smoking habits, *Clostridium* was the most significant feature obtained in BQC. *Acinetobacter* was observed to be the most significant among BQC having a chewing exposure of 0–10 years. In a previous study, the Actinobacteria phylum was reported to be the second most abundant oral bacterial phylum associated with betel nut chewing [31]. *Arcobacter* is a zoonotic food and water-borne pathogen, responsible for diarrhoea and known to harbor antibiotic and multi-drug resistance [72,73]. There could be an enhanced invasion in the oral cavity of BQC, due to an imbalance of beneficial microbes. *Aeromonas* and *Clostridium* have been reported to disturb the microbiome [74,75,79,80] *and* being associated with dental caries formation. Presence of *Clostridium* in the male smoker BQC corroborated with earlier studies claiming its involvement in oral carcinogenesis along with *Streptococcus*, *Prevotella*, and *Fusobacterium* [56,76,77]. Further focussed studies may discern the role of BQ chewing, smoking, and alcoholism-induced dysbiosis in oral cancer. Recent Studies are now exploring the role of probiotic organisms displaying anti-tumor and antimicrobial activities [78,79]. This approach of consumption of probiotics as an aid to reverse the effects of long-term BQ chewing effect exerted on the oral microbiome and to delay the deleterious effects of the BQ chewing habit needs to be explored.

In BQC, *Lachnoclostridium* and *Cycloclasticus* were not classified up to species level. Other than these, *Desulfosarcina*, *Herbaspirillum* in BQNC, *Chelatococcus*, *Optitus*, and *Xanthomonas* were reported among BQOC. *Desulfosarcina*, a sulfate-reducing anaerobic bacterium is a probable resident of subgingival sites [80] which plays a role in hydrocarbon degradation in marine trenches [81]. Thus, it can be prospected for its role in ethanol metabolism in drinkers and metabolism of hydrocarbons in the oral cavity of smoker BQC. *Lachnoclostridium* is a common human gut bacterium displaying homeostatic and anti-inflammatory behaviour [74,82]. *Cycloclasticus* is generally found in the marine habitat which degrades polycyclic aromatic hydrocarbon (PAH) compounds aerobically [83]. This bacterium may be involved in the degradation of the polycyclic hydrocarbons present in tobacco-associated products like BQ, where areca nut releases such harmful chemicals during the roasting/ preparation phase or mastication [84]. These chemicals include nitrites from the conjoined effects of salivary and BQ inflow to give rise to areca-derived nitrosamines N-nitrosoguvacoline, N-nitrosoguvacine, and 3-methylnitrosamino-propionitrile, a probable cause for DNA damage [85–87]. In BQ preparations, polycyclic aromatic hydrocarbons get involved through the combination of areca nut, calcium hydroxide (responsible for alkalinity), nicotine (from tobacco), flavorants, and colouring agents influencing the oral bacteria which play an important role in nitrate-nitrite-nitric oxide homeostasis [88].

BQ chewing has been linked to cancers and is a prevalent habit in India and other Asian countries as part of cultural practices and due to other reasons. The role of microbiome is emerging in various diseases including cancers. The present study has highlighted differences in oral microbiome in betel quid chewers and non-chewers with reference to its role in progression of oral diseases including cancer. The study was limited to the identification of alterations in the relative abundances of different bacterial communities in the BQ chewing and non-chewing population (n = 22) in the Delhi region of India. Also, there may be a number of factors including diet, genetics which can affect the microbial composition at the time of sampling, Besides, there are limitations of the meta-omics technology including standard procedures and computational tools for accurately analyzing the data stating a drawback of the 16s metagenomic approach. Further analysis for pathways will enable the identification of mechanisms to understand ways to re-establish homeostasis and regain a healthy state [89,90] in BQ chewing population.

## Supporting information

S1 Fig **a.** Phylum-level oral microbiome composition of whole population sampling (n = 22): 14 phyla including Firmicutes (38%), Proteobacteria (29%), Bacteroidetes (25%); **b.** Relatively Abundant phyla detected in (n = 22) on the basis of OTU.
(TIF)

S2 Fig **a.** Genus-level oral microbiome composition of whole population sampling (n = 22): 78 genera including *Prevotella* (15%), *Bacteroides* (12%), *Streptococcus* (11%), *Neisseria* (10%) and *Haemophilus* (9%); **b**. Relatively abundant genera detected in (n = 22) based on OTU assigned and (>1%) threshold.
(TIF)

S3 Fig **a.** Genus-species level oral microbiome composition of whole population sampling (n = 22): 75 species including *Prevotella denticola* (17%), *Streptococcus mutans* (11%), *Neisseria anima*lis (10%), Bacteroides dorei (8%), and *Haemophilus parainfluenzae* (6%); **b.** Relatively abundant genera detected in (n = 22) based on (>1%) threshold and OTU assigned.
(TIF)

S4 Fig **a.** Genus-unclassified species-level taxonomy for the oral microbiome of whole population sampling (n = 22): 30 Genus-unclassified species including *Bacteroides* (21%), *Streptococcus* (14%), *Acetobacter* (12%), *Prevotella* (10%), *Haemophilus* (8%), and *Neisseria* (6%); **b.** Relatively abundant genera-unclassified species detected in (n = 22) on the basis of OTU assigned.
(TIF)

S5 Fig PCoA plot: **a.** Chewers Vs. Non-Chewers; **b.** Age-group (21–30 years) Chewers Vs. Non-Chewers.
(TIF)

S6 Fig PCoA plot: **a.** Males Chewers Vs. Non-Chewers; **b.** Associated substances: Chewers Vs. Alcohol and smoking chewers.
(TIF)

S7 Fig PCoA plot: **a.** Associated substances: Chewers Vs. Smoker chewers; **b.** BQ Chewing history (0–10 years Vs. 10 years and above.
(TIF)

S8 Fig Most Significant Features Comparison with lowest p-value of **a.** Betel quid chewers Vs. Non-Chewers; **b.** (21–30 years old young individuals) Betel quid chewers Vs. Non-Chewers.
(TIF)

S9 Fig Most Significant Features Comparison with lowest p-value of **a**. Betel quid (Males) Chewers Vs. Non-Chewers; **b.** Betel quid Chewers Vs. Alcoholic-Smoker Chewers.
(TIF)

S10 Fig Most Significant Features Comparison with lowest p-value of **a**. Betel Quid Vs. Smoker Chewers; **b.** Betel Quid Chewer (Chewing History).
(TIF)

S11 Fig Relative abundance displayed for *Prevotella*- chewers, *Bacteroides*- non-chewers, and *Streptococcus*- Occasional chewers. (TIFF)

S12 Fig Unique elements in between chewers and Non-chewer abundant microbial community. (TIF)

S13 Fig Alpha Diversity pattern: **a.** Box and whisker plots of diversity and richness of microbiome Diversity analyzed through the Observed Genus diversity index. Dark horizontal lines represent the median, the box plots- the first (Q1) and third (Q3) quartiles, the outer fences- interquartile range, and the circles outside- outliers. **b.** Evenness index of Observed genus plots, explaining higher evenness among BQ chewer and non-chewer individuals.
(TIF)

S14 Fig Comparative representation of most abundant phylum in reference to NCBI and eHOMD database. (TIF)

S15 Fig Whisker-Box plots representation for Phylum Proteobacteria, based on relative abundance expressed according to **a.** NCBI database, **b.** eHOMD database.
(TIF)

S16 Fig Whisker-box plots representation for Phylum Firmicutes, based on relative abundance expressed according to **a.** NCBI database, **b.**) eHOMD database.
(TIF)

S17 Fig Workflow for data filtration and processing. (TIF)

S1 Graphical abstract (TIF)
