# Supplementary figures and images for: Identifying oral microbiome alterations in adult betel quid chewing population of Delhi, India
Source: PLoS One. 2023 Jan 4;18(1):e0278221. doi: 10.1371/journal.pone.0278221 (PMC9812331; doi:10.1371/journal.pone.0278221)

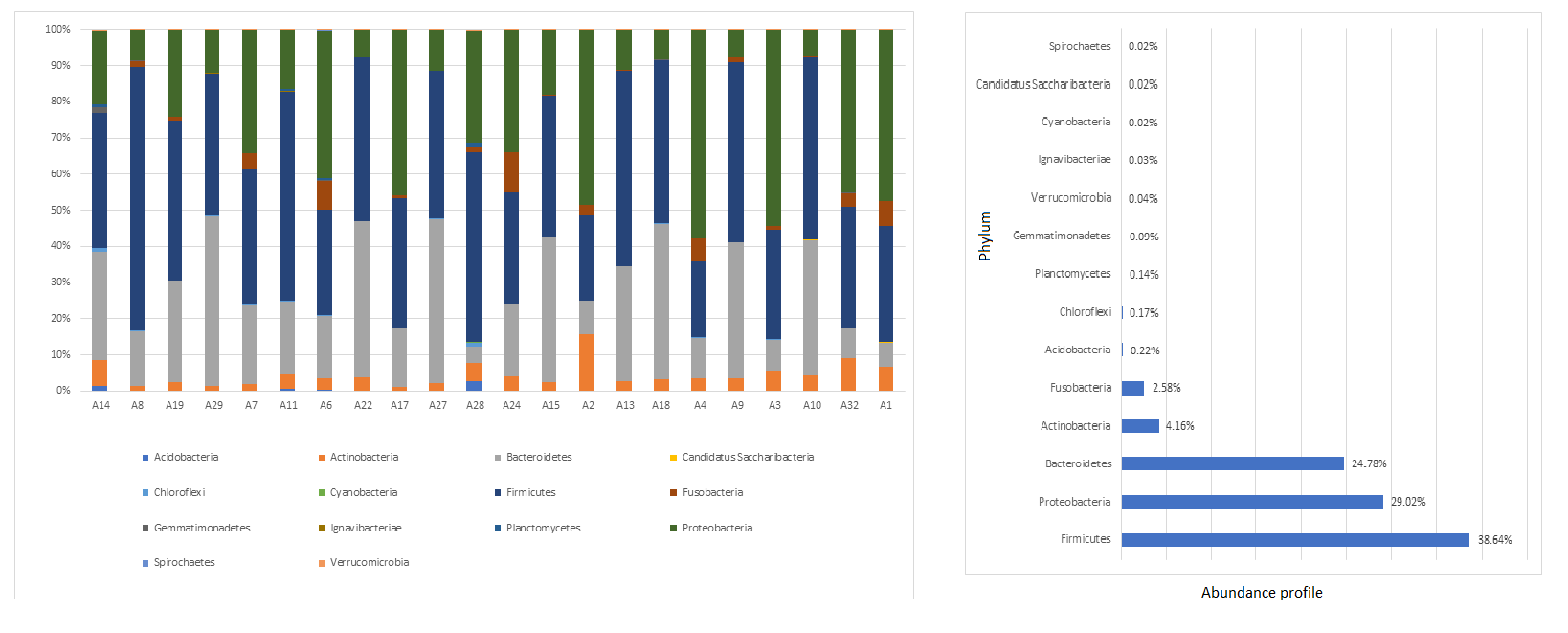

Supplement: S1 Fig — a. Phylum-level oral microbiome composition of whole population sampling (n = 22): 14 phyla including Firmicutes (38%), Proteobacteria (29%), Bacteroidetes (25%); b. Relatively Abundant phyla detected in (n = 22) on the basis of OTU. (TIF) [file pone.0278221.s001.tif]

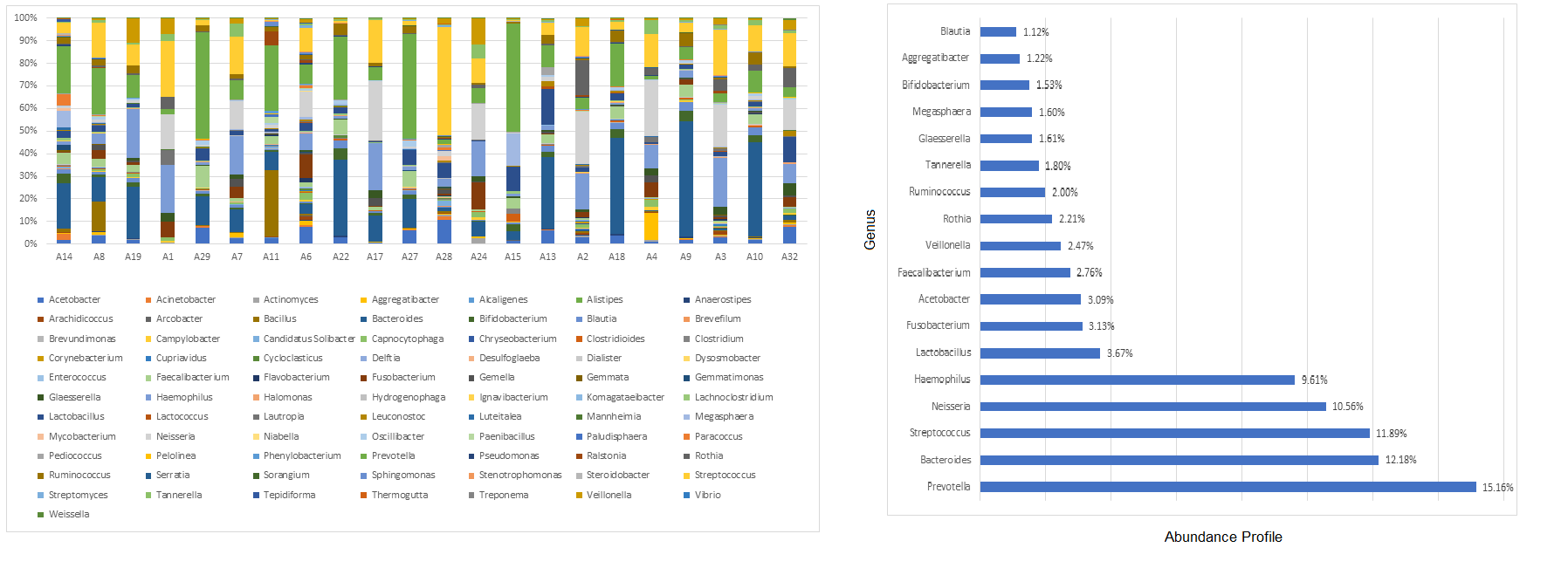

Supplement: S2 Fig — a. Genus-level oral microbiome composition of whole population sampling (n = 22): 78 genera including Prevotella (15%), Bacteroides (12%), Streptococcus (11%), Neisseria (10%) and Haemophilus (9%); b. Relatively abundant genera detected in (n = 22) based on OTU assigned and (>1%) threshold. (TIF) [file pone.0278221.s002.tif]

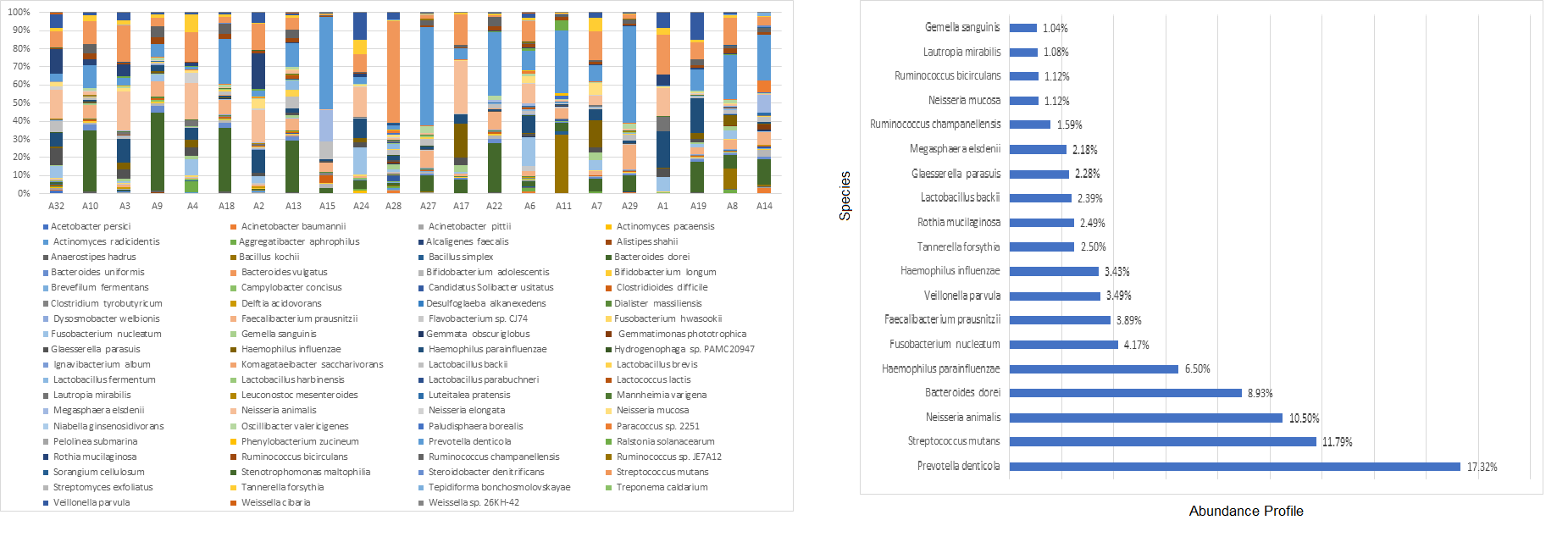

Supplement: S3 Fig — a. Genus-species level oral microbiome composition of whole population sampling (n = 22): 75 species including Prevotella denticola (17%), Streptococcus mutans (11%), Neisseria animalis (10%), Bacteroides dorei (8%), and Haemophilus parainfluenzae (6%); b. Relatively abundant genera detected in (n = 22) based on (>1%) threshold and OTU assigned. (TIF) [file pone.0278221.s003.tif]

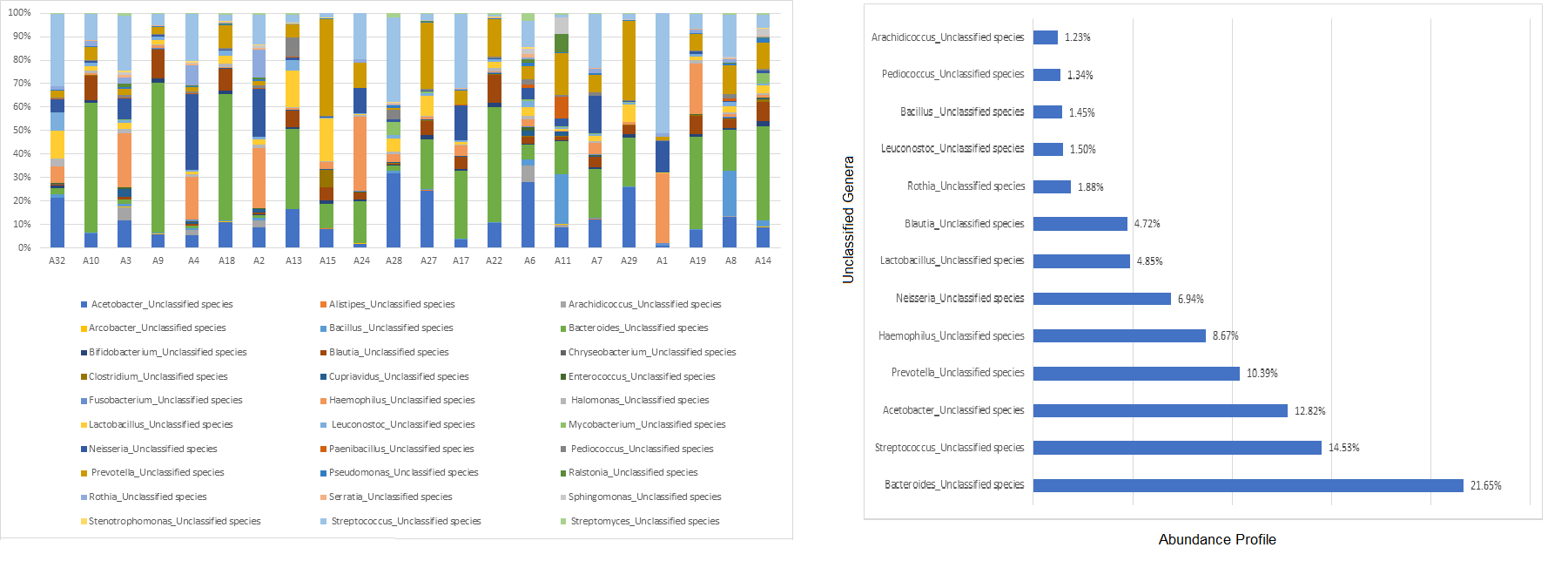

Supplement: S4 Fig — a. Genus-unclassified species-level taxonomy for the oral microbiome of whole population sampling (n = 22): 30 Genus-unclassified species including Bacteroides (21%), Streptococcus (14%), Acetobacter (12%), Prevotella (10%), Haemophilus (8%), and Neisseria (6%); b. Relatively abundant genera-unclassified species detected in (n = 22) on the basis of OTU assigned. (TIF) [file pone.0278221.s004.tif]

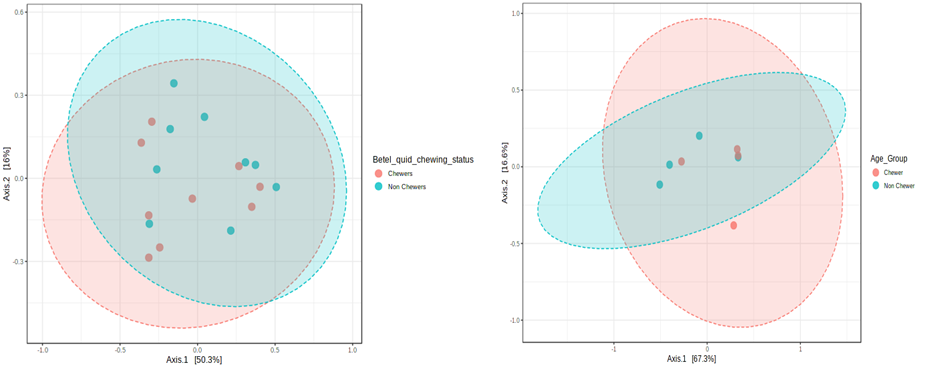

Supplement: S5 Fig — PCoA plot: a. Chewers Vs. Non-Chewers; b. Age-group (21–30 years) Chewers Vs. Non-Chewers. (TIF) [file pone.0278221.s005.tif]

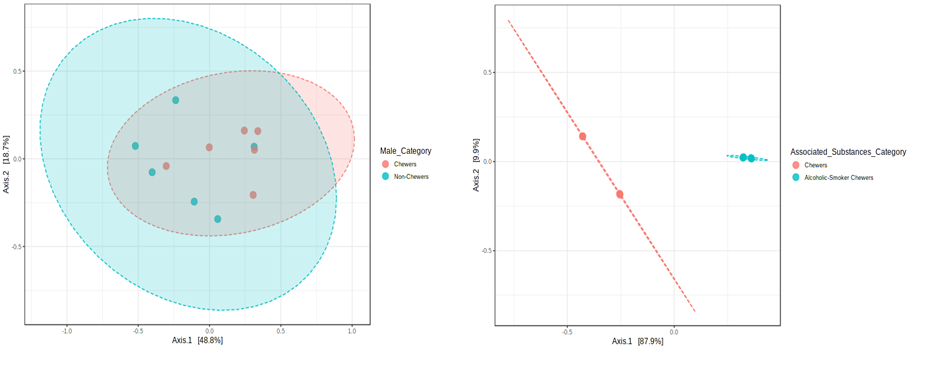

Supplement: S6 Fig — PCoA plot: a. Males Chewers Vs. Non-Chewers; b. Associated substances: Chewers Vs. Alcohol and smoking chewers. (TIF) [file pone.0278221.s006.tif]

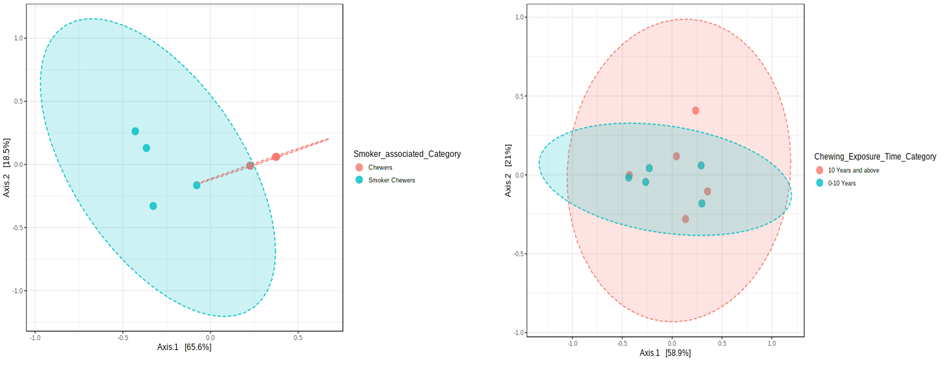

Supplement: S7 Fig — PCoA plot: a. Associated substances: Chewers Vs. Smoker chewers; b. BQ Chewing history (0–10 years Vs. 10 years and above. (TIF) [file pone.0278221.s007.tif]

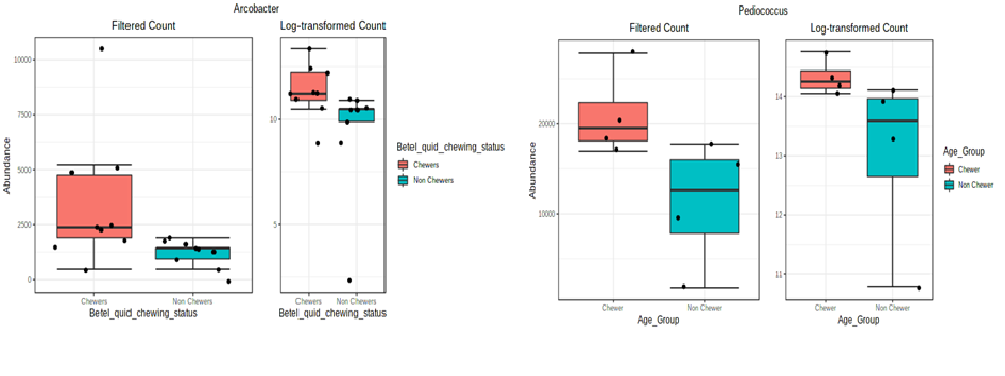

Supplement: S8 Fig — Most Significant Features Comparison with lowest p-value of a. Betel quid chewers Vs. Non-Chewers; b. (21–30 years old young individuals) Betel quid chewers Vs. Non-Chewers. (TIF) [file pone.0278221.s008.tif]

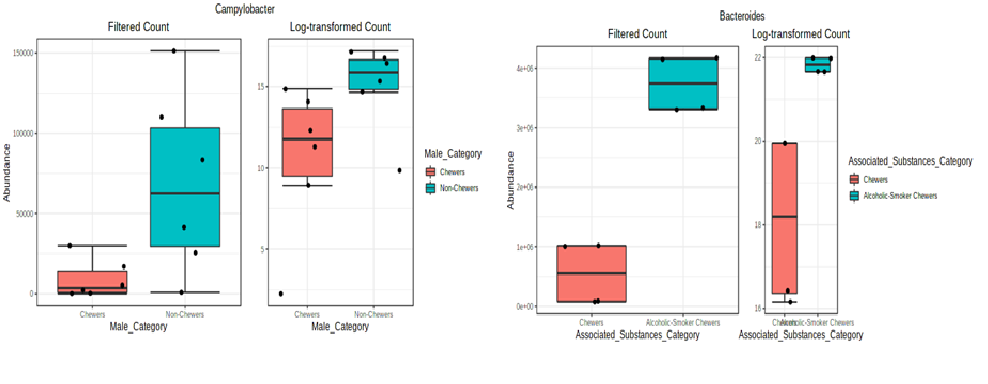

Supplement: S9 Fig — Most Significant Features Comparison with lowest p-value of a. Betel quid (Males) Chewers Vs. Non-Chewers; b. Betel quid Chewers Vs. Alcoholic-Smoker Chewers. (TIF) [file pone.0278221.s009.tif]

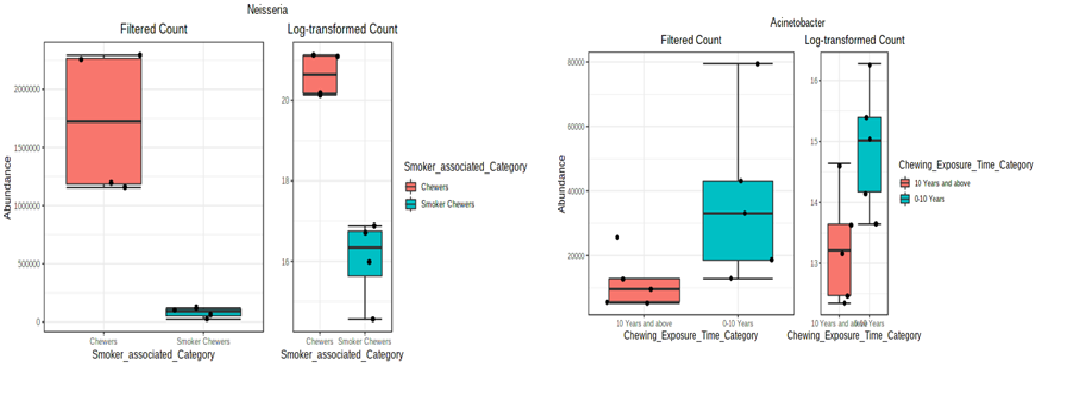

Supplement: S10 Fig — Most Significant Features Comparison with lowest p-value of a. Betel Quid Vs. Smoker Chewers; b. Betel Quid Chewer (Chewing History). (TIF) [file pone.0278221.s010.tif]

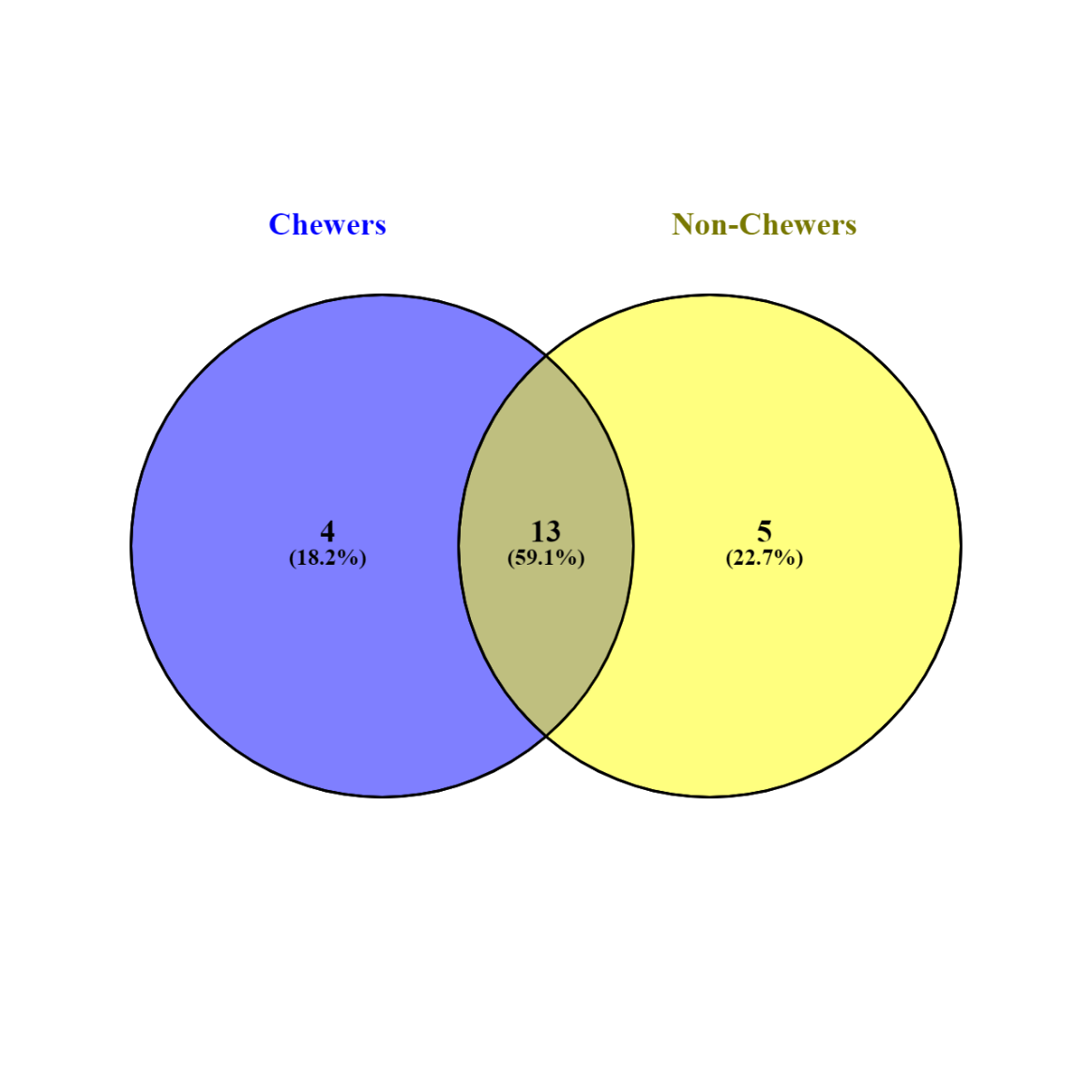

Supplement: S12 Fig — (TIF) [file pone.0278221.s012.tif]

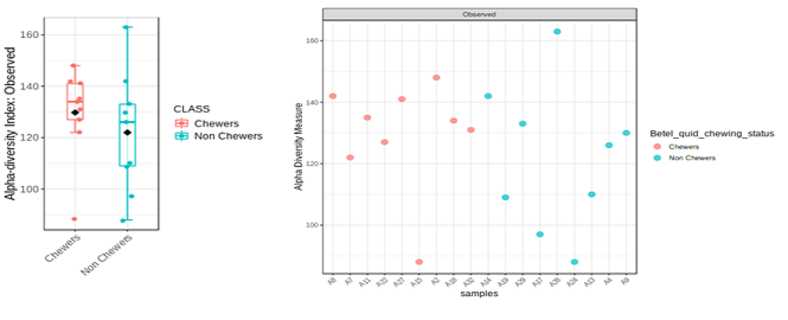

Supplement: S13 Fig — Alpha Diversity pattern: a. Box and whisker plots of diversity and richness of microbiome Diversity analyzed through the Observed Genus diversity index. Dark horizontal lines represent the median, the box plots- the first (Q1) and third (Q3) quartiles, the outer fences- interquartile range, and the circles outside- outliers. b. Evenness index of Observed genus plots, explaining higher evenness among BQ chewer and non-chewer individuals. (TIF) [file pone.0278221.s013.tif]

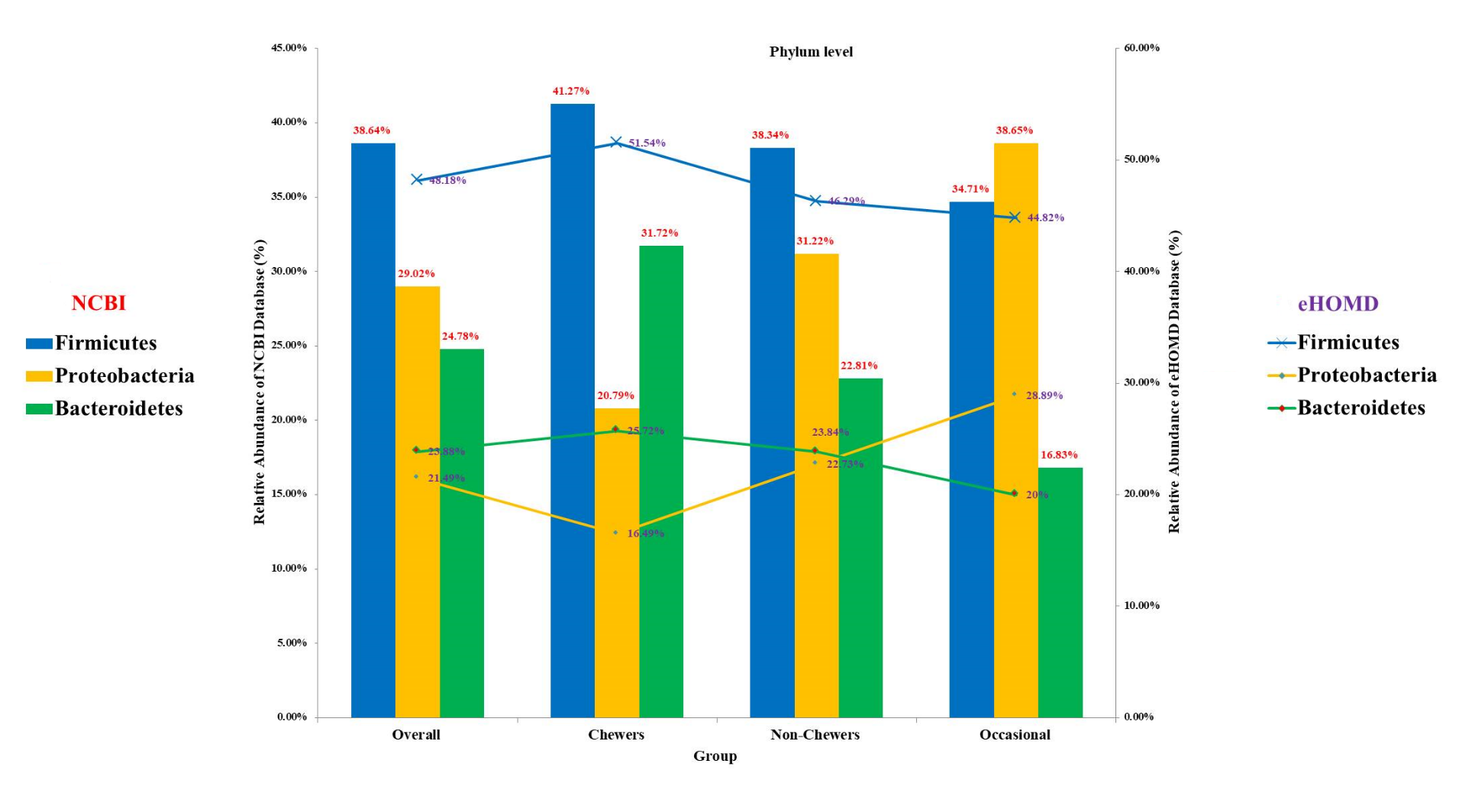

Supplement: S14 Fig — (TIF) [file pone.0278221.s014.tif]

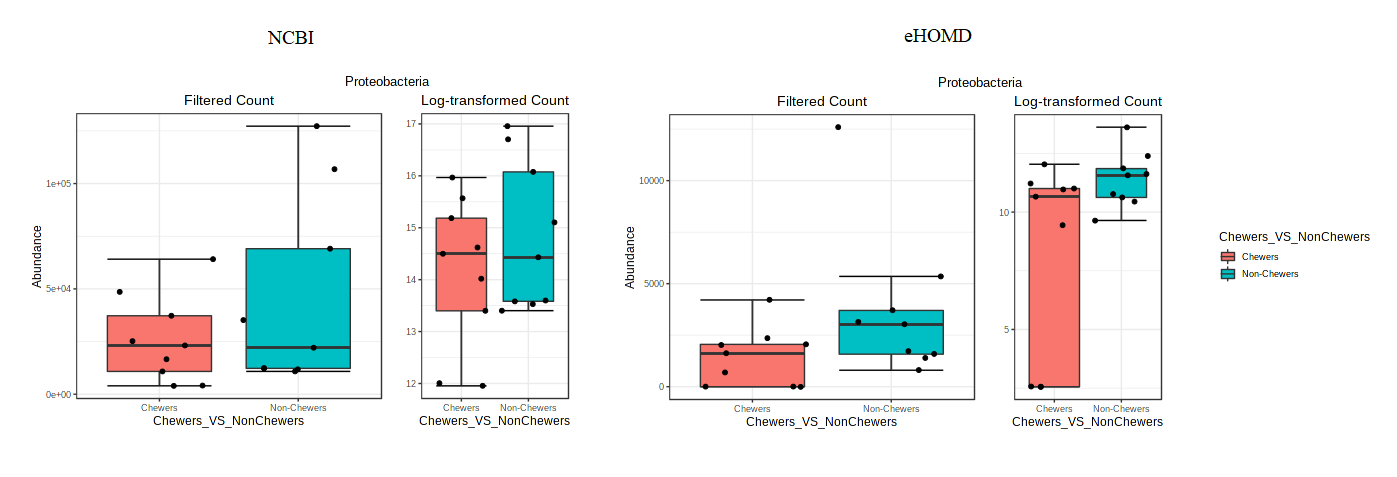

Supplement: S15 Fig — Whisker-Box plots representation for Phylum Proteobacteria, based on relative abundance expressed according to a. NCBI database, b. eHOMD database. (TIF) [file pone.0278221.s015.tif]

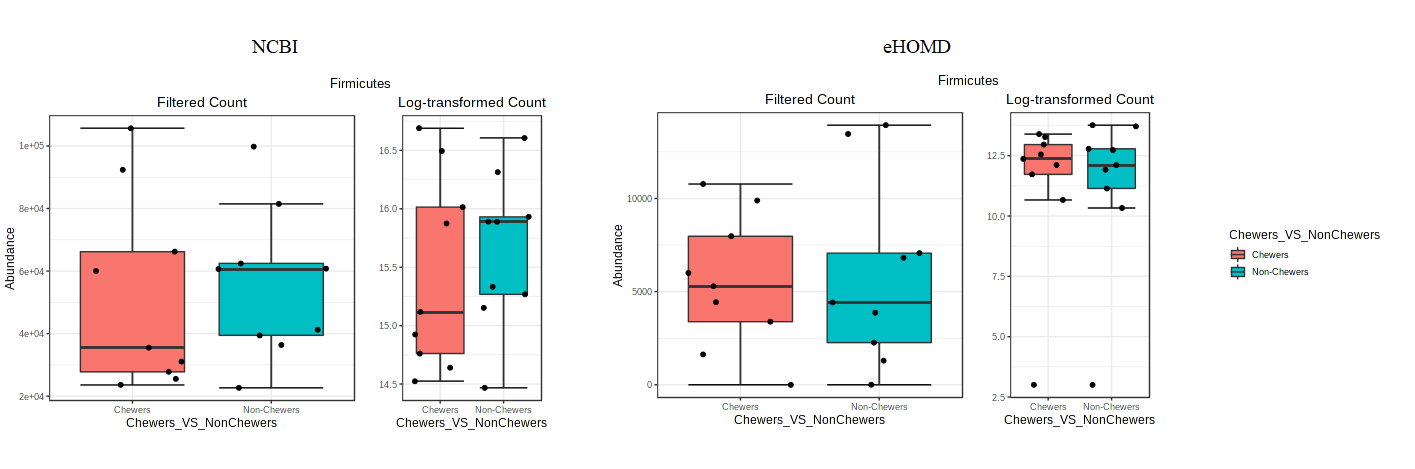

Supplement: S16 Fig — Whisker-box plots representation for Phylum Firmicutes, based on relative abundance expressed according to a. NCBI database, b.) eHOMD database. (TIF) [file pone.0278221.s016.tif]

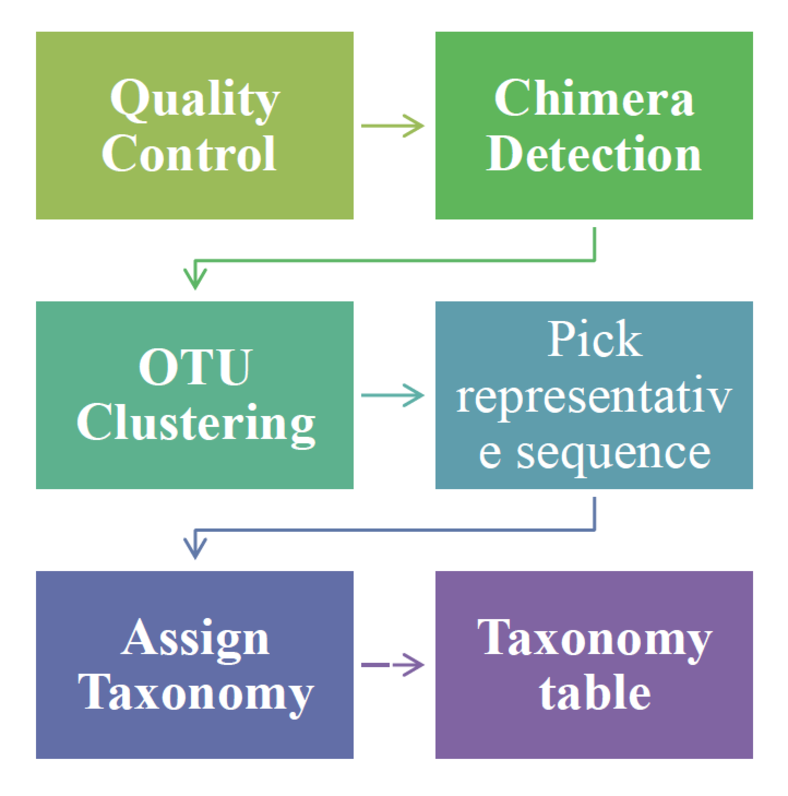

Supplement: S17 Fig — (TIF) [file pone.0278221.s017.tif]

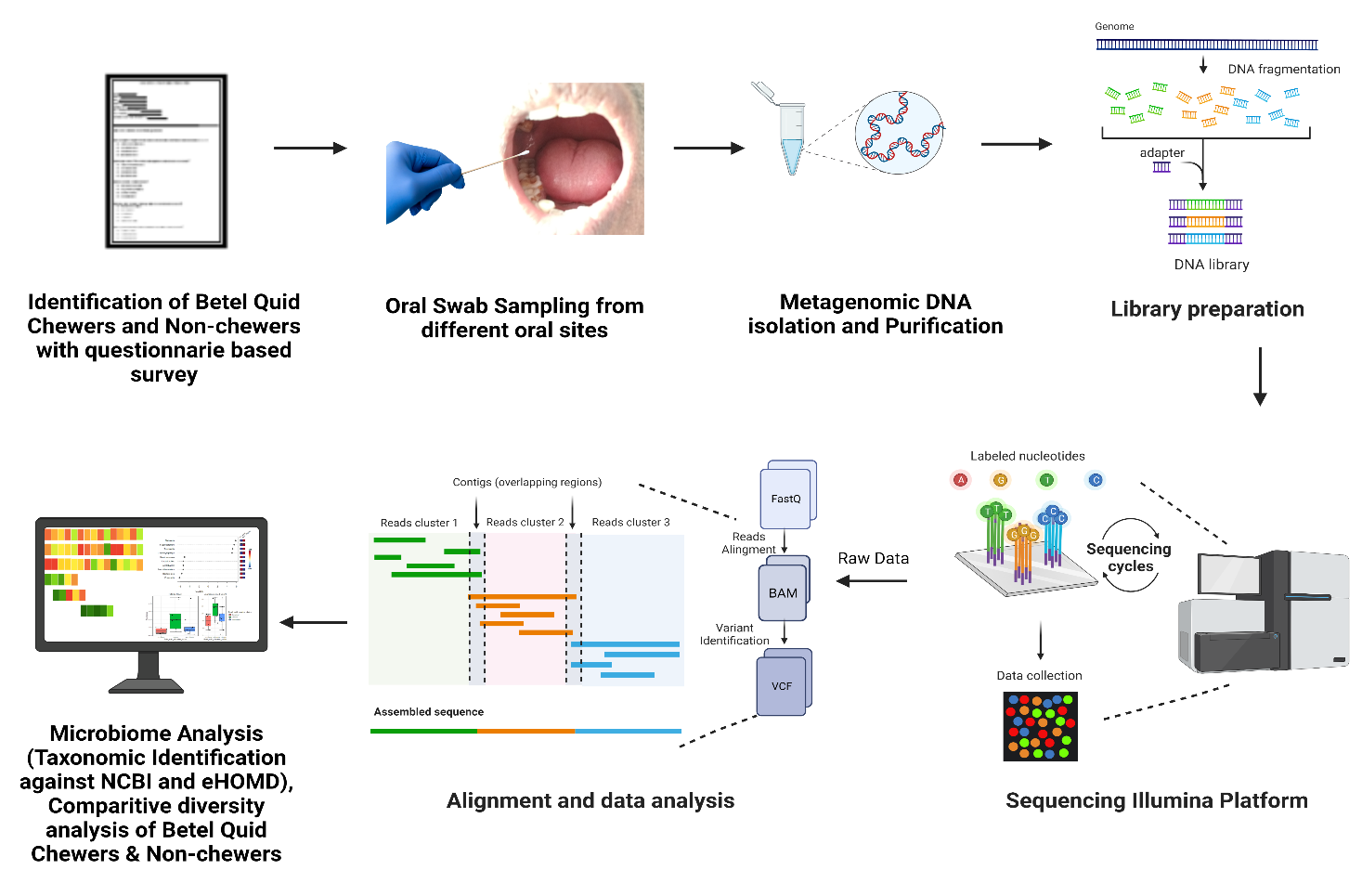

Supplement: S1 Graphical abstract — (TIF) [file pone.0278221.s018.tif]
